# Emergency deployment of direct air capture as a response to the climate crisis

**DOI:** 10.1038/s41467-020-20437-0

**Published:** 2021-01-14

**Authors:** Ryan Hanna, Ahmed Abdulla, Yangyang Xu, David G. Victor

**Affiliations:** 1 Center for Energy Research, University of California San Diego, La Jolla, CA 92093 USA; 2 Deep Decarbonization Initiative, University of California San Diego, La Jolla, CA 92093 USA; 3 Department of Mechanical and Aerospace Engineering, Carleton University, Ottawa, ON K1S 5B6 Canada; 4 Department of Atmospheric Sciences, Texas A&M University, College Station, TX 77843 USA; 5 School of Global Policy and Strategy, University of California San Diego, La Jolla, CA 92093 USA; 6 Scripps Institution of Oceanography, University of California San Diego, La Jolla, CA 92093 USA; 7 The Brookings Institution, Washington, D.C. 20036 USA

**Keywords:** Climate-change mitigation, Climate-change mitigation, Climate-change policy, Climate-change mitigation

## Abstract

Though highly motivated to slow the climate crisis, governments may struggle to impose costly polices on entrenched interest groups, resulting in a greater need for negative emissions. Here, we model wartime-like crash deployment of direct air capture (DAC) as a policy response to the climate crisis, calculating funding, net CO_2_ removal, and climate impacts. An emergency DAC program, with investment of 1.2–1.9% of global GDP annually, removes 2.2–2.3 GtCO_2_ yr^–1^ in 2050, 13–20 GtCO_2_ yr^–1^ in 2075, and 570–840 GtCO_2_ cumulatively over 2025–2100. Compared to a future in which policy efforts to control emissions follow current trends (SSP2-4.5), DAC substantially hastens the onset of net-zero CO_2_ emissions (to 2085–2095) and peak warming (to 2090–2095); yet warming still reaches 2.4–2.5 °C in 2100. Such massive CO_2_ removals hinge on near-term investment to boost the future capacity for upscaling. DAC is most cost-effective when using electricity sources already available today: hydropower and natural gas with renewables; fully renewable systems are more expensive because their low load factors do not allow efficient amortization of capital-intensive DAC plants.

## Abstract (web summary)

Governments may struggle to impose costly polices on vital industries, resulting in a greater need for negative emissions. Here, the authors model a direct air capture crash deployment program, finding it can remove 2.3 GtCO_2_ yr^–1^ in 2050, 13–20 GtCO_2_ yr^–1^ in 2075, and 570–840 GtCO_2_ cumulative over 2025–2100.

## Introduction

With the 2015 Paris Agreement, there were hopes that governments had finally turned the corner toward serious action on climate warming. However, even before the global pandemic, actual cuts in emissions lagged far behind Parisian ambition^1–3^: emissions have been rising at 1–2% per year^4^ and the gap between emissions and what is needed to stop warming at aspirational goals like 1.5 °C is growing^5^. To stabilize warming at 1.5 °C^6^, studies find that societies must remove previously-emitted CO_2_ from the atmosphere using negative emission technologies (NETs)^7–9^ or otherwise significantly curtail energy use^10^. The global pandemic has cut emissions temporarily, but historical patterns suggest they will rebound^11^. Indeed, much of the economic stimulus during the pandemic has focused on incumbent industrial activities, though Europe is a notable exception.

In response to these realities, the dialogue on climate policy is shifting—away from measured and technocratic policy designs, such as steadily rising carbon taxes and energy efficiency standards, and toward much graver warnings of emergency^12,13^. Dozens of national governments representing nearly 300 million people, over 1000 local administrative governments, and scores of scientists^14^ have made formal declarations of a climate crisis that demands a crisis response. With growing evidence of impacts attributable to climate change, a planet that is warming faster than expected^15^, and political pressures that are shifting quickly as well, it is imperative that scholars build a field of research that examines how a crisis mindset might affect climate policy. In times of crisis, such as war or pandemics, many barriers to policy expenditure and implementation are eclipsed by the need to mobilize aggressively around new missions^16^—often in ways that reinforce existing interest groups such as industrial producers. This logic of crisis politics suggests that the climate crisis may open new spigots of public spending but do little to weaken entrenched interest groups that have often impeded costly policy action. Ironically, big emitting industrial practices could remain in place even as societies becomes increasingly agitated about climate change and willing to spend massively on solutions.

Here we elaborate the parameters for one possible element of crisis response: a crash program to deploy direct air capture (DAC) systems that remove CO_2_ from ambient air. We build on recent reviews^17^, economic assessments^18,19^ and deployment scenarios^20,21^ of DAC. Policy responses forged with the politics and mindset of a crisis could involve numerous components—for example, massive spending on deployment of low-emission technologies and deep energy efficiency, among many others. Our purpose here is to elaborate one component, DAC, that might also prove attractive. Though public attitudes vary^22^, from a technological and industrial perspective DAC has attributes of high value to the politics of emergency response: deployments are modular, scalable, and highly controllable by the governments and firms that invest; carbon removals are verifiable; and deployment does not inherently harm existing industrial interests. Though energy intensive, DAC appears to have no biophysical limits^23^, unlike bioenergy with carbon capture and sequestration (BECCS). Nor does DAC require large-scale land use changes and hence compete with important sustainability goals such as maintaining biodiversity and food production^24,25^. Moreover, unlike strategies for controlling emissions from industry and the broader economy, deploying DAC does not intrinsically require intrusive policy interventions, such as requiring existing firms to transform their production methods. History and theory show that such industrial transformations are challenging to do quickly^26^, not least when they must be implemented simultaneously in many countries so that leaders do not suffer harm to their economic competitiveness^27^. Territorial control is an attribute of acute political importance because it allows nations, even unilaterally, to take domestic action that can have global impact. In the past, much attention has focused on unilateral action with regard to solar geoengineering^28–30^; an analogous literature for less emotive NETs is overdue.

In this paper we assess the potential for an emergency DAC deployment program to slow and reverse the rise in atmospheric CO_2_ and global mean temperature. The novelty of our experiment requires a new, integrated modeling framework to represent interactions between the three main components of an emergency program. First, we estimate the financial resources that might be available for emergency deployment, grounding that in political theories of crisis decision-making. Second, we build a bottom-up deployment model that constructs, operates, and retires successive vintages of DAC plants, given available funds and the rates at which DAC technologies might improve with experience. Such a model must include constraints on the speed with which novel industries can scale and must also characterize the costs and emissions from the many types of energy supplies (heat and electricity) that could power DAC. Third, we link the political and techno-economic modeling of the first two components to climate models that estimate the effects of these deployments on the carbon cycle, atmospheric CO_2_ concentration, and global mean surface temperature.

In addition to novel methods, four new insights emerge from our analysis. First, crisis-level funding—on par with spending during major wars but sustained over longer time horizons—would create a fund in excess of one trillion U.S. dollars per year for spending on DAC. Second, in this crisis funding mode the constraints on DAC deployment in the 2–3 decades following the start of the program are not money but scalability. For policy makers, one implication of this finding is the high value of near-term DAC deployments—even if societies today are not yet treating climate change as a crisis—because near-term deployments enhance future scalability. Rather than avoiding DAC deployments because of high near-term costs, the right policy approach is the opposite. This finding poses challenges for modeling because scalability, while an essential concept, is hard to assess. This paper, for the first time in the DAC literature, deals squarely with the implications of scaling. Third, despite an emergency DAC program that removes prodigious amounts of CO_2_ (multiple gigatonnes annually by 2050 and rising substantially thereafter), concurrent deep mitigation of emissions, equivalent to SSP1-2.6, is still required to meet the Paris goal of limiting warming to 2 °C. Crisis deployment of DAC, even at the extreme of what is technically feasible, is not a substitute for conventional mitigation. Fourth, a fully decarbonized supply of electricity is not a prerequisite for cost-effective DAC. DAC is most cost-effective when paired with energy supplies that already exist today—such as electric grids comprised predominately of hydropower, or combined cycle gas power plants, or gas with a growing share of renewables. Fully renewable systems are significantly more expensive because their low load factors do not allow efficient amortization of capital-intensive DAC plants. Regardless, the near-term political approach to crash deployment should seek not to maximize CO_2_ removals but rather to deploy many plants to push the technology down the learning curve—which does not require fully decarbonized energy supplies.

## Results

### A framework for integrated analysis

**Fig. 1:**
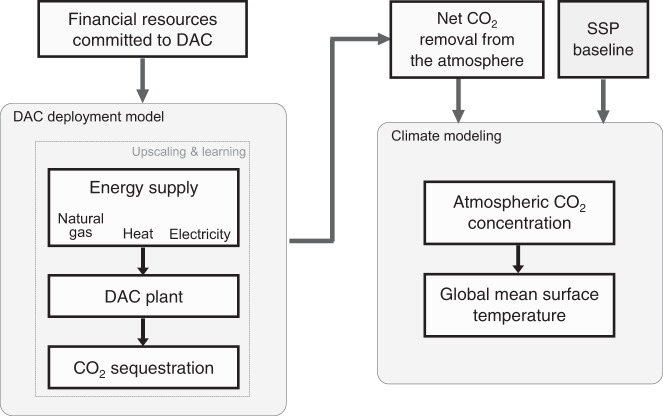
Conceptual schematic of the modelling framework. The model calculates impacts on the climate system due to DAC deployment and comprises three main parts: (1) an estimate of the financial resources available to fund DAC; (2) deployment of a fleet of DAC plants, including requisite supplies of electricity and heat, and upscaling of these DAC-energy system combinations over time; and (3) the impact of these DAC-energy systems on atmospheric CO_2_ concentration and global mean surface temperature, given background emissions which are taken from the shared socioeconomic pathways (SSP). (See Supplementary Fig. 1 for a more detailed schematic).

DAC deployment depends centrally on three factors: funding made available, choice of DAC process, and choice of energy supplies needed to power DAC. We build a model that captures the interactions among these (Fig. 1; Methods). The choice of DAC process, and its potential for improvement through experience, affects how many plants can be built within a fixed investment budget. The attributes of energy systems—cost and availability—have similar effects. And the question of how much DAC experience money buys affects, in turn, rates of learning that depend on deployment.

Following the logic of international relations, we envision three modes of crisis deployment differentiated by degree of cooperation^27,31,32^. The simplest is unilateral action: a single nation allocates a percentage of its GDP annually to DAC deployment and thus signals to its populace (and the world) that it is serious about climate. Here we consider a large democracy, the United States, that devotes 5% of GDP—similar in scale to sustained historical wartime mobilization^33^, although expenditures during peak years are much higher^34^. (A unilateral EU deployment program would yield similar results because the European economy is similar in size to the $20 trillion U.S. economy.) The second mode considers cooperation amongst a club of motivated democracies^35^, whose governments plausibly have strong incentive to provide solutions to a climate crisis as their polities demand conspicuous action and mete electoral punishment on leaders who fail^36^. We consider the OECD (Organization for Economic Cooperation and Development), a club of 36 democracies that emerged over six decades from the effort to consolidate democratic governance and economic growth in Europe after the Second World War. In our rendering, an OECD-like club allocates a portion of member GDPs for DAC deployment, with shares proportional to today’s OECD budget contributions. The third mode envisions global cooperation, with cost sharing along familiar lines: IBRD (International Bank for Reconstruction and Development) members contribute funding according to their contribution to the IBRD budget. The three funding regimes yield an enormous, crisis-level resource base: initial appropriations total $1 trillion, $1.4 trillion, and $1.6 trillion per year, respectively (Supplementary Tables 1–4).

We expect that the club of democracies funding model is most likely—a group of countries, each too small to generate decisive impact independently, find common cause. The approach is exactly analogous to that used by alliances that go to war or provide collective security—while the incentives for collective action vary by country, the common incentive compels the coalition of the willing^35^.

Despite the massive resources that might become available when a nation or group acts in crisis, industries invariably face constraints on the rate at which they can scale to absorb new funds efficiently. We reflect this reality by limiting the number of deployable plants at the program’s outset to five. (In our analysis DAC plants have a capacity of 1 MtCO_2_ yr^–1^ gross capture, reflecting current ambition and practice. For comparison, just one DAC plant of this size is being organized today.) We also enforce a maximum industry-wide growth rate, which we set to 20% per year—as used in a recent IAM study^21^ of DAC. Industry growth rate is a critical unknown that merits further investigation. The history of photovoltaics—a highly-modular, globally-marketed product that over 2007–2018 saw annual growth of >25% in every year and 46% CAGR (compound annual growth rate; IEA PVPS)—suggests that much higher rates are attainable. So too does the U.S. Liberty ship building program^37^—a crash U.S. Government Second World War program that rapidly scaled over its initial 12 months (monthly growth rates all >20%) to achieve an overall 92% CAGR over 4 years. The French nuclear program, orchestrated and funded by a national government highly motivated to reduce dependence on imported energy, saw annual growth rates of 12–41% in 8 of 11 years during its major phase of construction (1977–1987); it achieved 16% CAGR over that period, but growth relaxed to <10% over the next decade. The historical record is replete with varied rates and forms of technology diffusion^38^, and the trajectory that DAC could experience is uncertain and unclear. We use 20% annual growth as a first-order estimate that balances the historical evidence—and note that it may well be too conservative for individual years yet too optimistic for a program sustained over multiple decades.

Commercial firms have piloted two promising DAC processes—each with distinct requirements for heat and electricity, as well as cost. We model both: a solid sorbent system of the type under development by the firm Climeworks and a liquid solvent system of the type pursued by the firm Carbon Engineering^18^ (Table 1). The former requires low-temperature heat (<150 °C), making it feasible to use waste heat from industrialized applications, while the latter needs higher temperatures (~900 °C) that demand dedicated supplies. We consider variants of the liquid solvent system that source heat from oxy-fuel gas combustion, electric heating, and hydrogen combustion, which are discussed extensively in a recent consensus report^19^ by the U.S. National Academies of Sciences; and we consider the solid sorbent system coupled to combustion, heat pump, and waste heat sources—in total, six unique configurations (Supplementary Table 5).

**Table 1:** Initial (floor) assumptions for energy demand and cost of DAC processes considered in this analysis.

DAC process	Heat requirement	Electricity demand (kWh tCO_2_^–1^)	Heat/gas demand (GJ tCO_2_^–1^)	Capital cost (2018$ tCO_2_^–1^ yr^–1^)	Reference
LT	<150 °C	444 (286)	4.8 (3.4)	2170 (812)	ref. ^19^
HT-gas	~900 °C	366 (366)	5.3 (5.3)	1053 (729)	ref. ^18^
		594 (350)	12.2 (5.3)	1334 (722)	ref. ^19^
HT-electric	–	4358 (3322)	–	769 (592)	ref. ^19^
HT-hydrogen	–	5497 (3244)	–	2112 (1120)	ref. ^19^

The LT DAC process is defined by demand for heat generally; HT DAC is defined by demand for natural gas. Data are available from commercial firms^18^ and academic sources^19^, which we distinguish throughout this investigation. (See Supplementary Table 6 for full data sets of modeled DAC processes.)

*LT* low-temperature, *HT* high-temperature.

We model all plausible combinations of electricity and heat supply because each varies in cost, carbon intensity, and availability. For heat, we consider combustion with and without carbon capture and storage (CCS), waste heat, and heat pumps (Supplementary Table 7). For electricity, we consider the dominant modes of electric power generation that exist today or that might plausibly exist in a low-carbon future: hydropower, combined cycle gas turbines (CCGT) with and without CCS, renewables with and without storage, and small modular nuclear reactors (SMRs). We also model hybrids of renewables, CCGT, and storage—a technology portfolio that reflects the direction of evolution of many power systems (Supplementary Tables 8–10). Energy sources emit CO_2_, which is accounted in calculations of net CO_2_ removal (Methods), where net indicates gross removal less process emissions.

Unique combinations of funding regime, DAC process, and energy supply yield a total of 294 plausible configurations—what we call “scenarios” throughout.

The efficacy of DAC deployment is measured, ultimately, by its impact on climate. We use two climate models^39,40^ to calculate the capacity for deployment to draw down atmospheric CO_2_ and slow then reverse rise in global mean temperature. Climate impacts derive largely from net CO_2_ removal. We include fugitive methane emissions at 0.32% leakage rate, the collective 2017 average methane intensity of aggregated upstream gas and oil operations of Oil and Gas Climate Initiative members—the firms that have most conspicuously embedded methane control into their operations and are targeting cuts to 0.25% by 2025. Although the impact of fugitive methane emissions is small in our analysis—a direct result of our assumption of leakage rate—the problem is serious^41–43^. We expect that a crisis response leading to massive deployment of DAC would strictly enforce best practices for producing and transporting methane that might be needed to power the technology.

### Funding, expenditure and deployment

**Fig. 2:**
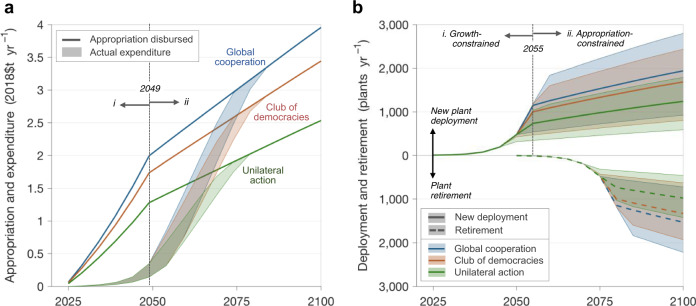
Funding and deployment by funding regime. **a** Funding disbursed for DAC deployment and actual expenditure on DAC. The appropriation disbursed for deployment (lines) is the sum of annualized disbursements from each yearly allocation. There are two distinct periods of disbursement: (i) prior to 2050, disbursements increase rapidly as, year after year, a new DAC vintage begins operation; (ii) post-2050, disbursements level off as the number of DAC vintages operating saturates. Actual expenditure (ribbons; 15th and 85th percentile scenarios) is the total appropriation ultimately spent, given industry’s capability to scale up to use funds. Variation in expenditure stems from the differing costs of DAC process and energy supplies used. **b** New DAC plant deployment and retirement. Ribbons indicate 15th and 85th percentile scenarios; lines show medians. Over 2025–2055 (i), deployment grows exponentially and at maximum rate and is thus growth-constrained. Post-2055 (ii), growth is fiscally constrained and linear; further increases in plant deployment stem from falling plant costs and an appropriation growing with GDP. The result is logistic (S-shaped) growth. (See Supplementary Figs. 6 and 7 for deployment and fleet size by scenario).

The three funding regimes produce appropriations totaling between $1 trillion and $1.6 trillion initially, which then grow with GDP. For several decades after initializing the deployment program (from 2025 to 2050–2060), available funding far exceeds industry’s ability to utilize those resources (Fig. 2a), due to the maximum industry growth constraint. During those years, unspent funding is forgone. Initially, funds are used entirely to build new plants; after 25 years, plants are retired and replaced (Fig. 2b). (We treat lifetime operating and maintenance costs as a sinking fund that is financed at the point of construction.) An important unknown is the real lifetime of a DAC plant. If it remains fixed, then around year 2050 the deployment program will require a complete one-for-one replacement of old plants. If, however, DAC plants show substantial opportunity for lifetime extension, as with modern nuclear plants, then the economics of the program could change radically.

### Net CO_2_ removal

**Fig. 3:**
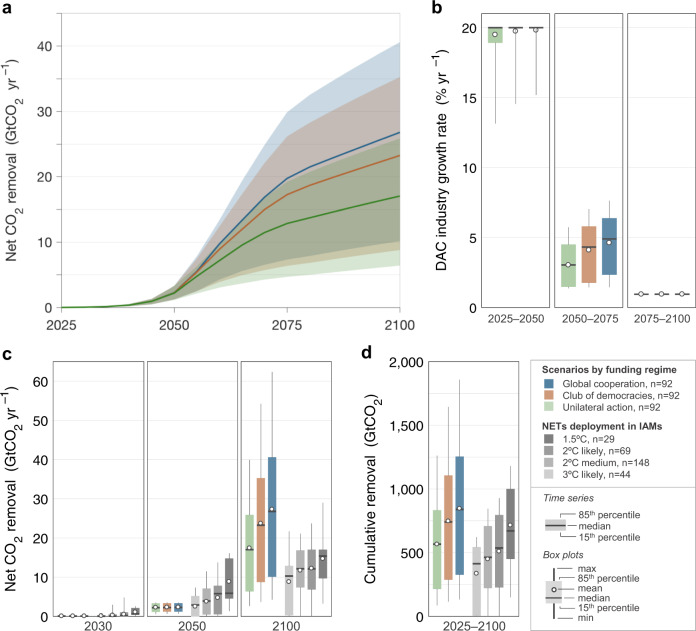
Net CO_2_ removal by funding regime. **a** Net CO_2_ removal (i.e., gross removal less process emissions from energy supply) over 2025–2100. Colors denote the funding regime; ribbons indicate the 15th and 85th percentile scenarios; solid lines show the median. **b** DAC upscaling rate, given as the compound annual growth in DAC plants by quarter-century. The maximum allowable growth rate in the model is 20% yr^−1^. **c** Net CO_2_ removal in this study compared to removal by NETs in IAM scenarios^17^. IAM scenarios, both with and without overshoot, are binned into four groups according to how they limit temperature rise in 2100: 1.5 °C scenarios limit warming to 1.5 °C with >50% probability; likely 2 °C scenarios, 2 °C with >66% probability; medium 2 °C scenarios, 2 °C with >50% probability; and likely 3 °C scenarios, 3 °C with >66% probability. See ref. ^17^ for assignments of SSP scenarios to these categories. **d** Cumulative net CO_2_ removal over 2025–2100 with analogous comparisons to IAM scenarios. (See Supplementary Fig. 5 for CO_2_ removal by individual scenario).

Answering the question of how quickly NETs can scale under real-world conditions is of pivotal importance in determining the feasibility and investment trajectories for meeting 1.5° or 2 °C warming goals. While IAMs have struggled with questions about plausible rates of NETs deployment^23,44,45^, a consensus expert review^17^ suggests DAC’s 2050 potential could be as high as 5 GtCO_2_ yr^–1^. By contrast, we find that even our extreme crash deployment program results in more modest levels of net removal from the atmosphere of 2.2–2.3 GtCO_2_ yr^–1^ in 2050 (median; 15–85^th^ percentile range, 1.2–3.3 GtCO_2_ yr^–1^; Fig. 3; Table 2). Similar to a recent IAM study of DAC^21^, the factor limiting scaleup through 2050 in our analysis is the growth rate (Fig. 3b); achieving 5 GtCO_2_ yr^–1^ in 2050 requires sustained growth of 25% per year (as we will show later).

**Table 2:** Net CO_2_ removal compared to published IAM deployment of NETs.

	2030	2050	2075	2100	Cumulative (2025–2100)
This analysis: Median (15–85^th^ percentile) net CO_2_ removal by DAC, GtCO_2_					
Unilateral U.S. action	0.05 (0.03–0.07)	2.2 (1.2–3.3)	12.9 (4.7–19.2)	17 (6.4–25.9)	566 (215–832)
Club of democracies: OECD	0.05 (0.03–0.07)	2.3 (1.2–3.3)	17.3 (6.4–26.2)	23.3 (8.8–35.3)	741 (288–1105)
Global cooperation	0.05 (0.03–0.07)	2.3 (1.2–3.3)	19.7 (7.3–29.9)	26.8 (10.1–40.6)	839 (328–1254)
By comparison: Median (15–85^th^ percentile) net CO_2_ removal by NETs in four categories of IAM scenarios, GtCO_2_					
3 °C likely	0 (0–0.3)	2.9 (0–5.2)	–	10.3 (0.3–12.9)	413 (5–543)
2 °C medium	0.1 (0–0.6)	3.9 (0.5–7.1)	–	12.1 (7.4–16.8)	464 (222–707)
2 °C likely	0.2 (0–1)	5.8 (0.6–7.8)	–	12.1 (6.9–17)	537 (227–795)
1.5 °C	0.6 (0.5–2.1)	5.9 (4.6–14.7)	–	15.4 (9.7–17)	671 (451–999)

IAMs deploy multiple NETs and have explored primarily BECCS to remove CO_2_; DAC has not been widely assessed. Results are a compilation from IAM databases (AMPERE, LIMITS, RoSE, SSP) and recent studies^7,9,59^ focused on achieving 1.5 °C. See the Fig. 3 caption (or ref. ^17^) for definition of 3 °C likely, 2 °C medium, 2 °C likely, and 1.5 °C scenarios; see ref. ^17^ for assignments of SSP scenarios to these categories.

In our analysis, large removals are achieved only after 2050, totaling 13–20 GtCO_2_ yr^–1^ in 2075 and 17–27 GtCO_2_ yr^–1^ in 2100 (range of median removals of the three funding regimes; Table 2). Removals increase rapidly over 2050–2075 (Fig. 3a) as 25 years of sustained investment bear fruit and large technological improvements through learning are realized. Median removals in 2100 in our model exceed the published NETs requirement of 1.5 °C scenarios of 15 GtCO_2_ yr^–1^ (10–17 GtCO_2_ yr^–1^ 15–85^th^ percentile range; Fig. 3c). In addition, cumulative removal in 2100 generally falls within published estimates of 1.5 °C and 2 °C scenarios (Fig. 3d).

The history of adoption of novel technologies suggests that scaleup requires decades^46–48^, and our analysis implicates the same. We find that achieving large CO_2_ removals is a protracted process, the observable impacts of which become salient only after multiple decades of maturation, even when enormous resources are on hand. The central goals of early deployment should include achieving technological maturity (e.g., through significant research and development) and increasing the scale of industry so that, later on, it can absorb the full available financial resource to maximize deployment rates.

### DAC’s impact on climate

We investigate the impact of CO_2_ removal on two scenarios from the IPCC’s Shared Socioeconomic Pathways (SSPs)^49^. Our goal is to calculate DAC’s impact on warming in a world that largely follows ongoing trends, in which efforts at collective mitigation fail to varying degrees—a world where emergency deployment of DAC is perhaps most plausible. Hence we choose SSP2, IPCC’s middle-of-the-road scenario^50^ in which social, economic, and technological trends follow historical patterns and moderate challenges to mitigation emerge.

One scenario is the marker SSP2, a future without collective global mitigation and with rising emissions. In this setting where collective mitigation continues to prove politically vexing, some governments might nonetheless be highly motivated to act on climate change. They could adopt policies that attempt to push other countries to mitigate—for example, aggressive trade and investment policies that carry big geopolitical risks—and in concert invest in DAC (or other NETs) along the extreme scenario we analyze in this paper. Countries may act unilaterally or in clubs to spend money on joint projects even as they find it difficult to achieve much global cooperation needed for highly intrusive, deep cuts in emissions. These DAC leaders would be the same countries that are already leading to cut their own emissions, but those cuts at home have little effect because these nations were already on a trajectory of lower emissions.

The other scenario is SSP2-4.5, in which mitigation is increased beyond efforts today but nevertheless falls far short of meeting Paris goals^51^. This second scenario is perhaps the most plausible pathway for the kind of extreme DAC effort we model here. Governments do what they can—with flaws in design and implementation and many holdout countries that do little and cannot be compelled with trade sanctions and other penalties to act further—and then invest massively in DAC to achieve more protection for the climate.

These pathways span a range of partial mitigation—from efforts that increase ambition yet fall short of meeting international goals to efforts that fail to arrest emissions altogether. They reflect plausible states of a world wracked by climate change, in which industrial politics in most countries are stuck, to varying degrees, dealing with entrenched interests and international cooperation is tenuous, making it hard for governments to justify imposing costly policies on local industries despite political interest in tackling the crisis. Mindful that there are many possible futures, in the SI we include analysis against a wider range of mitigation pathways, including a more optimistic outlook, SSP1-2.6 (ref. ^52^), and a more pessimistic one, SSP5-8.5 (ref. ^53^).

**Fig. 4:**
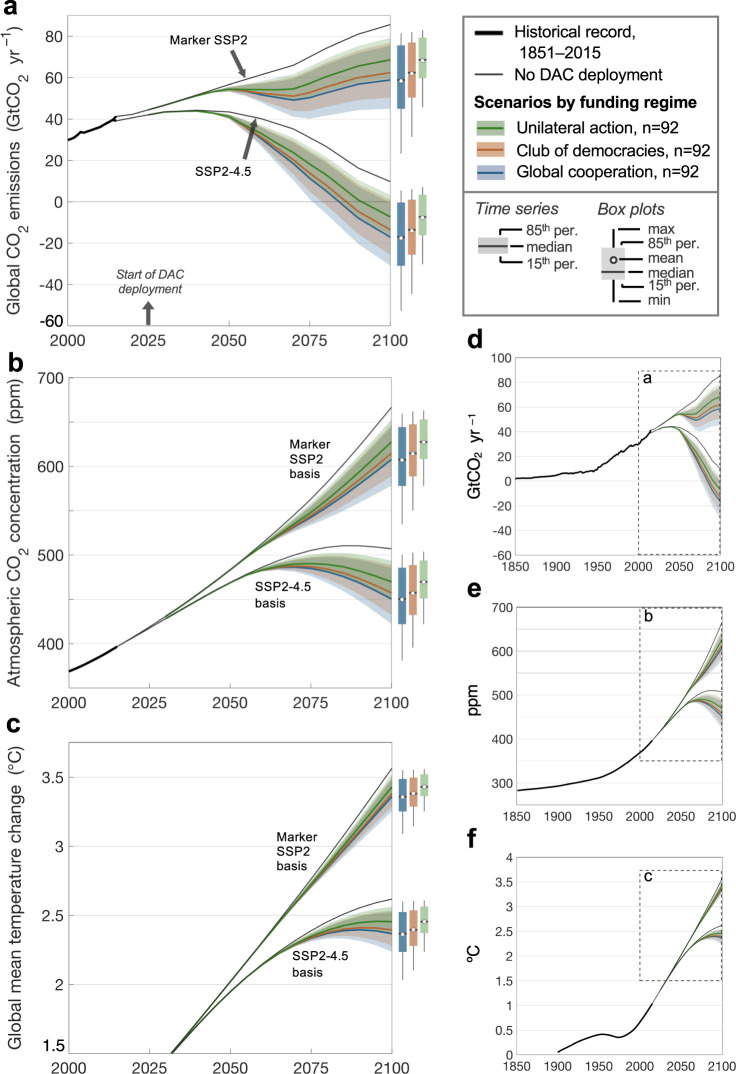
Climate benefits of net CO_2_ removal assuming marker SSP2 and SSP2-4.5 emission futures. **a** Global CO_2_ emissions. SSP emissions are from the SSP database version 2.0 (https://tntcat.iiasa.ac.at/SspDb; ref. ^49^). **b** Atmospheric CO_2_ concentration. **c** Global mean temperature change relative to pre-industrial levels (1850–1900). **d**–**f** Trends beginning in 1850. Ribbons indicate the 15th and 85th percentile scenarios; solid lines indicate the median. Black lines show the case of no DAC deployment. Boxes show the 15th and 85th percentile scenarios in 2100; center line, median; dot, mean; whiskers, range. (Here we show SSP2, IPCC’s middle-of-the-road pathway; we include scenarios with a wider range of emissions—SSP1-2.6 and SSP5-8.5—in Supplementary Fig. 8).

With the marker SSP2, median global CO_2_ emissions at end-of-century exceed 58 GtCO_2_ yr^–1^ despite massive CO_2_ removals (Fig. 4a). With SSP2-4.5, emissions peak at 44 GtCO_2_ yr^–1^ in 2040, even without DAC. DAC’s effect is to significantly decrease the magnitude and hasten the timing of peak CO_2_ concentration—from 510 ppm in 2090 to 486–490 ppm in 2070–2075 (Fig. 4b; median). By 2100, median concentrations are 451–470 ppm, a 42–63% reduction in atmospheric CO_2_ rise over 2025–2100 compared to the case without DAC, and trending downward.

The considerable inertia in the carbon cycle and climate system delays the temperature benefit of DAC beyond 2100. Despite demonstrable progress in reducing concentrations, SSP2-4.5 mitigation combined with crisis-level deployment of DAC results in global mean temperature rise of 2.4–2.5 °C in 2100 (Fig. 4c; median)—a 0.1–0.2 °C reduction (13–19%) in the warming that occurs over 2025–2100 without DAC (warming of 2.6 °C in 2100). Nevertheless, the effect of DAC on the temperature trajectory is substantial—it arrests the growth in the warming curve, which peaks at 2.4–2.5 °C in the 2090s. For model runs that extend further into the future, DAC reverses temperature rise to 1.9–2.2 °C in 2150, a reversal of 38–61% of the warming that occurs without DAC, which sees temperatures reach 2.7 °C in 2150 and rise even further thereafter.

Only with much greater ambition, such as the substantial mitigation in SSP1-2.6, do DAC CO_2_ removals help achieve the Paris goal of limiting temperature rise to well below 2 °C. With DAC and SSP1-2.6 mitigation, temperature rise peaks just under 2 °C in 2070. By 2100, it is 1.6–1.7 °C, which is 0.2–0.3 °C lower than the 1.9 °C temperature rise observed without DAC (Supplementary Fig. 8).

### Individual scenarios: cost and energy requirement

We compare the performance of different DAC system configurations in terms of cost, net CO_2_ removal, and energy use. Patterns in performance are similar across funding regimes, so we present results from one regime here: that of the club of democracies.

**Fig. 5:**
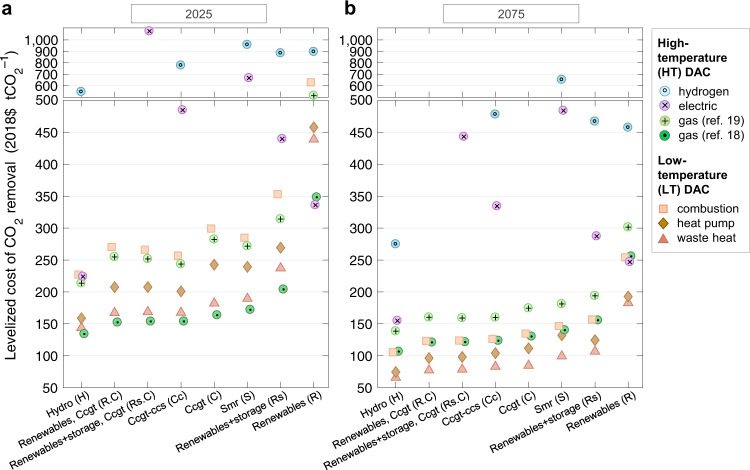
The marginal levelized cost of net CO_2_ removal (LCOR). Shown are years when the deployment program is nascent (2025; **a**) and mature (2075; **b**). Scenarios are shown by DAC configuration (markers) and electricity supply (*x*-axis). For configurations with energy storage, only the best-performing (lowest LCOR) scenario is plotted. For scenarios not shown, process CO_2_ emissions exceed removals. Funding is from the club of democracies. The *y*-axis breaks and changes scaling beginning at 500 2018$ tCO_2_^–1^. (We show LCOR in 2025 and 2075 to highlight major trends; Supplementary Fig. 9 shows results over 2025–2100. Supplementary Figs. 10 and 11 further show LCOR by component cost.).

Figure 5 shows the marginal levelized cost of net CO_2_ removal (LCOR) from the atmosphere—the ratio of a DAC plant’s lifetime cost to lifetime net CO_2_ removal. The LCOR is a marginal value that changes over time as the cost to build and operate falls and performance improves. Three distinct LCOR clusters emerge by DAC process: LT systems are lowest cost (66–254 2018$ tCO_2_^–1^ in 2075; Fig. 5b) followed by HT-gas systems (107–301), while HT-electric (155–484) and HT-hydrogen (275–653) are very expensive. HT-gas systems are initially cheapest (Fig. 5a) because they initially have lower capital expenditures than LT systems. After investment and consequent technological learning, LT systems perform better because they achieve lower capital costs than HT systems, and their energy demand is lower, too. These cost assessments follow directly from DAC system specifications, which are characterized by large uncertainties inherent in the preliminary technology assessments that currently characterize DAC. Another benefit of the LT systems is their ability to leverage alternative heat sources, including heat pumps and waste heat.

Technological learning emerges as crucial to every scenario, as evidenced by the decline in costs between 2025 and 2075 (Fig. 5). LCOR improvement is rapid, occurring primarily over 2025–2050 due to exponential growth in deployments over that time. Reductions in DAC capital and operating costs are responsible for 65–100% (88% on average) of the decrease in the total cost of LT and HT-gas systems, depending on scenario. Decreases in energy costs are responsible for the rest. Indeed, learning drives both HT and LT DAC systems to their lowest capital costs in 2040–2050 and 2060–2065, respectively, though learning rates are uncertain as well.

Distinct clusters similarly emerge by energy supply. In general, existing low-cost and low-emission energy sources, such as hydroelectric power, yield lowest LCOR (66–139 2018$ tCO_2_^–1^ in 2075 for LT and HT-gas systems). Hydropower’s potential for expansion is quite uncertain, however. Next-best are hybrid gas and fully gas grids: CCGT (85–175), CCGT-CCS (83–160), renewables with storage and CCGT (79–159), and renewables with CCGT (77–160). These options outperform renewable supplies (183–301 without storage, 107–194 with storage) and SMRs (99–181). Gas is competitive despite process emissions (Supplementary Fig. 12) because of lower costs and higher utilization (Supplementary Fig. 13). The main disadvantage of renewables is their low utilization factor (uptime); they require costly investments in storage to increase utilization. SMRs perform poorly because they are expensive.

**Fig. 6:**
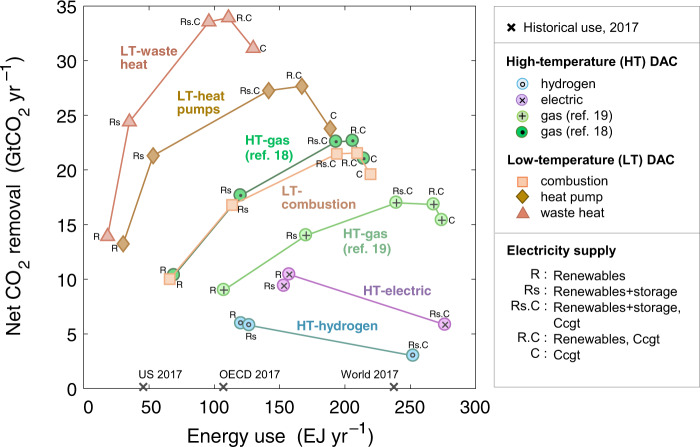
Net CO_2_ removal and energy use. Shown are the subset of scenarios with electricity supplies based on gas and renewables (with and without storage), which we find to be the most plausible; excluded are hydropower (significant challenges to scalability) and SMRs and CCGT with CCS (costly and unproven). The year shown is 2075, the program’s 50th year. Configurations are denoted by markers, with like-configurations (i.e., DAC processes with common heat sources) connected with lines to aid visualization. For scenarios with energy storage, only the best-performing (lowest 2075 LCOR) scenarios are reported. Historical 2017 energy use (electricity plus gas) is marked along the *x*-axis for reference. Funding is from the club of democracies. Electricity labels are consistent with Fig. 5. (For results over 2050–2100 see Supplementary Fig. 14. For disaggregation of energy use by electricity and gas see Supplementary Fig. 15.).

Figure 6 shows net CO_2_ removal and the total energy consumed to attain that removal for various DAC configurations. Compared with energy consumption in the IPCC’s marker SSPs^49^, HT-gas and LT DAC energy use reaches 9–14% of global electricity use in 2075 (median; 5–30%, 15th/85th percentiles) and 53–83% of global gas use in 2100 (median; 0–470%, 15th/85th percentiles). Operationally, LT systems are least energy-intensive, particularly those that utilize waste heat, which significantly reduces the need for new costly heat supply, or those coupled with heat pumps, which are efficient heat generators. Next-best are HT-gas systems. Were one to assume that waste heat could not be easily collected at scale or that heat pumps might not perform reliably, then LT and HT-gas systems perform nearly identically. By contrast, HT-electric and HT-hydrogen are energy intensive and thus also have low net CO_2_ removals (curves in lower-right quadrant of Fig. 6); moreover, coupling these systems with CCGT causes process emissions to exceed removals.

All energy supplies, no matter the type, would need to be scaled up in concert with the growing DAC fleet. If dedicated solely to DAC, existing CCGT capacity in the U.S., for example, could support removal of roughly 4–8 GtCO_2_ yr^–1^; hydropower, 0.5–1 GtCO_2_ yr^–1^; and renewables with storage, 0.9–2 GtCO_2_ yr^–1^, with variation in removal due to variation in DAC efficiency (kWh-consumed per tCO_2_-removed). SMRs and CCGT with CCS have not been deployed commercially at any meaningful scale.

### Critical uncertainties: upscaling and cost

**Fig. 7:**
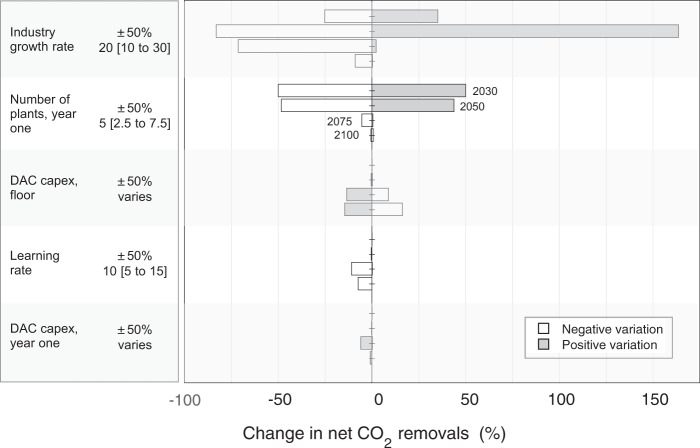
Net CO_2_ removal sensitivity to upscaling parameters, DAC costs, and learning rate. Bars show the mean change across scenarios in net CO_2_ removal in 2030, 2050, 2075, and 2100 due to variation in the single parameter. Parameters (nominal value and plus/minus variation) are listed at left. White bars show the effect of negative variation (e.g., decreasing growth rate or cost) and gray bars show positive variation. Funding is from the club of democracies. (For results on all key parameters see Supplementary Figs. 16 and 17.).

Crisis deployment, being both rapid and sustained, means that scaleup speed and long-term DAC costs are the determinative factors governing CO_2_ removal (Fig. 7). Having varied all key model parameters to plausible extremes, we find that the single most important source of sensitivity in our results is the industry growth rate—the annual rate at which the DAC fleet can grow. The number of plants deployed initially is also critical because growth stems from this anchor deployment. Initial costs matter much less than potentials for scaling and technological learning. Learning rates, if high, will eclipse modest variations in initial cost. Estimates for maximum performance (i.e., the performance ceiling for learning) have larger impact still—because DAC systems quickly achieve maximum performance during a precipitous scaleup period, while the bulk of deployment, which occurs later in the program, benefits from this early learning.

**Fig. 8:**
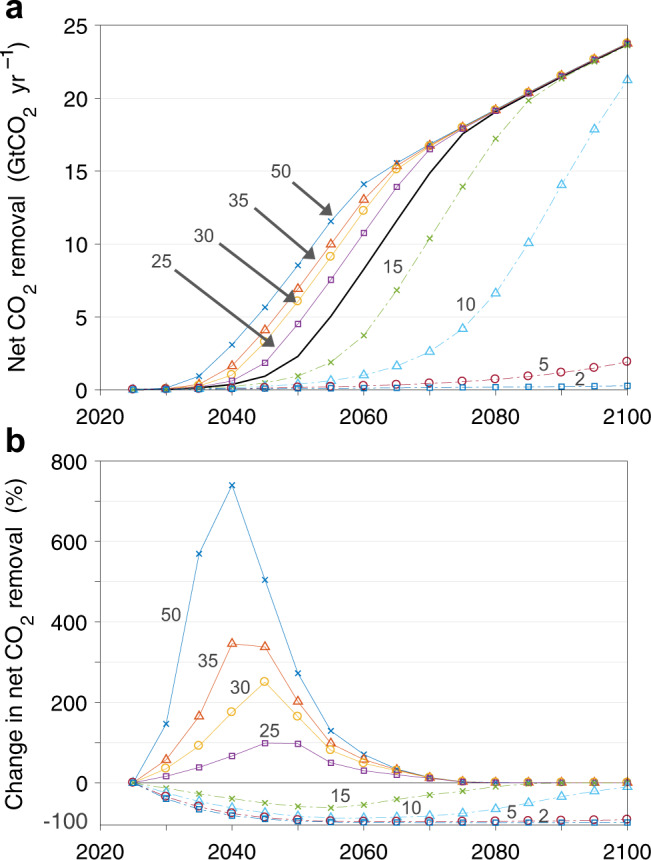
Net CO_2_ removal sensitivity to industry growth rate. **a** Net CO_2_ removal given variation in the maximum allowable industry growth rate, given in percent per year. Markers show the mean of all scenarios by year. The nominal growth rate (20% yr^−1^) is denoted with an unmarked black line; varied growth rates are denoted with marked colored lines and labels. **b** Percent change in net CO_2_ removal relative to the nominal case. Markers show the mean of all scenarios by year. Funding is from the club of democracies.

Industry growth rate is a vitally important yet unknown factor. In Fig. 8 we look at a wider range of growth rates, extending the analysis from 10–30% yr^–1^ (in Fig. 7) to 2–50% yr^–1^. As expected, relaxing the scalability constraint beyond our central estimate of 20% yr^–1^ allows substantially more net CO_2_ removal over 2030–2050 relative to the baseline (Fig. 7b); it also brings forward the date at which the industry growth rate no longer constrains deployment. With a growth rate of even 25% yr^–1^, removals in 2050 are doubled compared to the base case, from 2.3 GtCO_2_ to 4.5 GtCO_2_, and the growth rate stops constraining deployment in 2050 on average, compared to 2055 in the base case. Restricting the growth rate, on the other hand, seriously inhibits upscaling and extends the time required to reach milestones like 5 GtCO_2_ yr^–1^ removal (Fig. 8a). The rapid drop-off in CO_2_ removal when growth rates are constrained to just 10% yr^–1^ illustrates the central challenge of transitioning DAC from an early-stage, immature industry to one that, as promptly as possible, can utilize whatever resources society is willing to spend.

## Discussion

Emergency deployments are important to examine because they bound what may be feasible; they provide a measure of what could be achievable if societies choose to respond to the climate crisis with commensurate crisis mobilization. This approach to deployment is, moreover, consistent with the likely politics of crisis decision-making, which will emphasize spending for new deployments rather than actively taking on existing interest groups.

We find that the impact of DAC on net CO_2_ emissions and concentrations could be substantial—reversing rising concentrations beginning in 2070–2075 (Fig. 4). However, that reversal requires coincident mitigation equivalent to at least SSP2-4.5. Even with massive DAC deployment, substantial levels of remaining emissions in SSP2-4.5 lead to warming of 2.4–2.5 °C at the end of the century. Under scenarios of higher remaining emissions (marker SSP2), median warming in 2100 reaches 3.4 °C even with an emergency crash program for DAC. Sustained investment over 25 years with essentially unlimited funds sees deployment achieve 2.2–2.3 GtCO_2_ yr^–1^ in 2050 (Fig. 3)—with constraints on growth (i.e., scaleup) the limiting factor.

Though DAC costs dominate, choice of energy supplies materially affects cost (Fig. 5). While use of hydropower helps systems achieve lowest marginal cost, absent advances in the ability to scale hydropower or utilize waste heat, the economically best performing DAC systems are those that rely on natural gas—either through fully gas systems or gas-renewable hybrids (Supplementary Figs. 9–11, 13 and 14).

In terms of sheer numbers of DAC plants, all deployment scenarios involve massive buildout (Fig. 2b). HT-gas and LT DAC fleets total 800 plants in 2050, 3920–9190 in 2075, and 5090–12,700 in 2100 (Supplementary Figs. 6 and 7). These require a substantial, several-fold expansion of today’s global energy supply (Supplementary Fig. 15)—in many scenarios doubling global 2017 gas use and increasing electricity use by 50% in 2100. With such an expansion, DAC emerges as a new, major component of the global energy ecosystem: in 2075, it consumes 9–14% of global electricity use, and in 2100 it consumes 53–83% of global gas use.

Any such analysis with technologies that are immature today and involve decades of deployment will rest on many assumptions whose proper values are unknowable ex ante. Such uncertainties multiply into large spreads in estimated performance and cost of plants, energy requirements, and net CO_2_ removals. Future work must focus on strategies to anchor these assumptions and to understand how the unknowns affect optimal and real-world testing and deployment of DAC systems as part of a larger climate management strategy.

Our work offers at least three broad implications for DAC deployment strategy, with concomitant uncertainties that must be understood better. First, because crisis deployment is both rapid and sustained, initial costs matter much less than potentials for learning through deployment and maximum performance. One consequence of this perspective, as well, is that the choice of DAC process and energy supply option matter greatly, not least because each has a different performance ceiling that affects system costs and maximum net CO_2_ removal. Another consequence of this perspective is that the focus, today, on estimating levelized costs of removing CO_2_ (LCOR) for different DAC processes is too narrow. LCOR may affect which technologies are chosen for initial deployment, but what really matters for the long term are learning potentials and performance ceilings.

Second, our analysis (and any real-world deployment of DAC) is highly dependent on the maximum rate at which a DAC industry can scale over the first three decades of deployment (Figs. 7 and 8, Supplementary Figs. 16 and 17). Improved understanding of those maximum early rates of upscaling could help guide more aggressive crash programs, for instance by bounding the range of investment that would be needed in a new, immature technology like DAC, thereby freeing funds for other crisis solutions. Policy and analytical attention are needed, as well, on methods for assessing efficient scaling—that is, the rate at which prodigious funds available for construction can be used effectively. A close analysis of that question would benefit from attention to what has been learned by studying wartime mobilization^34^.

Third, it is vitally important to study DAC systems not as stand-alone technologies but as one component of the larger system. A systems approach forces one to look beyond the “obvious” choice that energy supplies for DAC should be low- or zero-carbon, or the assumption that these energy sources could cater to massive DAC deployment. Our study finds that the all-renewables systems perform poorly in cost-effectiveness and gross CO_2_ removal from the atmosphere, and that use of natural gas increases cost-effectiveness. More work is needed to study optimal configurations of gas, renewables and other elements of energy systems. For example, economically optimal DAC deployment programs might begin by utilizing hydro and gas-fired electricity, which have low marginal costs and high availability. Such early, lower-cost deployments can maximize learning effects before shifting to scalable renewable energy supplies (Supplementary Figs. 18 and 19).

A systems perspective applies, as well, to politics. The motivation for crash deployment is emergency-style politics, which we have articulated in the form of three types of possible funding programs. More help from political science is needed, including the study of international cooperation, to understand how perceptions of crisis might motivate responses by political leaders, along with the roles of different international alliances that could help share the cost. Whether deployments of DAC (or other NETs) are indeed more likely responses than aggressive collective mitigation of emissions needs investigation. An additional matter for investigation with the tools of political science is whether the geophysical attributes of NETs make them good candidates for emergency action. We have shown here the protracted delays between near-term expenditures and distant benefits; those delays might be unacceptable during political emergencies. Instead, other approaches with more proximate benefits, such as solar radiation management, might become more attractive.

Beyond these three questions, there are many others that concern system configuration and location-specific deployment—for example, land requirements and availability, potential limitation on geological pore space for sequestering CO_2_, the size of early utilization markets for captured CO_2_, and public acceptance of these engineering solutions. Others still concern timing and the consequences of delaying deployment (Supplementary Table 14).

It is time to extend research on DAC (and NETs generally) to the real-world conditions and constraints that accompany deployment—especially in the context of acute political pressures that will arise as climate change becomes viewed as a crisis. More practical analyses are necessary in an industry that is pregnant with potential, especially as the scale of the climate crisis becomes fully appreciated.

## Methods

### Configurations and deployment scenarios

We model six unique DAC plant configurations, where a configuration is a pairing of DAC process and heat supply (Supplementary Tables 5–7). Three of these configurations involve high-temperature (HT) liquid solvent process coupled to kilns that employ oxy-fired natural gas (HT-gas or “HT-g”), electricity (HT-electric or “HT-e”), or hydrogen (HT-hydrogen or “HT-h”). Three configurations are comprised of low-temperature (LT) solid sorbent process coupled to either a natural gas combustion-fired boiler (LT-g), a supply of waste heat (LT-w), or electric heat pumps (LT-hp). Geothermal heating, actively considered in some places^54^, is location-constrained, while our focus is scalable options. Extant pilot plants have been sized <1000 tCO_2_ yr^–1^; the plants considered in this analysis are of commercial size with capacity 1-MtCO_2_ yr^–1^. Hardware, fuel use, and carbon flows differ by configuration (Supplementary Figs. 3 and 4), as do the equations governing the system. We track differences by defining six disjoint sets that distinguish scenarios *s* by their configuration, i.e., $s \in {\cal{S}}_{{\mathrm{HT}}-{\mathrm{g}}} \cup {\cal{S}}_{{\mathrm{HT}}-{\mathrm{e}}} \cup {\cal{S}}_{{\mathrm{HT}}-{\mathrm{h}}} \cup {\cal{S}}_{{\mathrm{LT}}-{\mathrm{g}}} \cup {\cal{S}}_{{\mathrm{LT}}-{\mathrm{w}}} \cup {\cal{S}}_{{\mathrm{LT}}-{\mathrm{hp}}}$. DAC deployment modeling is implemented in Matlab.

Deployment scenarios are formed by pairing each of the six configurations with one of several non-proximate electricity sources along with an appropriation regime that funds deployment. Electricity supplies include renewables, hydroelectric power, CCGT, CCGT with CCS, SMRs, or hybrids thereof (Supplementary Tables 8–10). An appropriation is the amount of funding made available for deployment. Each scenario—294 in total—is a combination of DAC type, heat source, electricity source, and appropriation.

The modeling period *t* = {2025, 2030, ..., 2105} runs through end-of-century and is defined by *T* = 16 periods lasting Δ = 5 years. Results are, in general, denoted with vectors *v*_*i*_ of length *T* or matrices *m*_*i,k*_ of size T-by-T. Results can vary by period *k* ∈ {1, …, *T*} and by DAC vintage *i* ∈ {1, …, *T*}, where a vintage is the set of plants deployed (brought into operation) in period *t*_*i*_. Plants operate over the period $t_{{\mathrm{{\Omega} }}_i}$, where ${\Omega} _i = \{ i, \ldots ,i + L{\mathrm{/}}{\Delta} - 1\}$ and *L* is the plant operating lifetime in years.

### Appropriation

The appropriation for DAC deployment in period *t*_*k*_ is given by $A_k = A_1{\Delta} \mathop {\prod}\nolimits_{u = 1}^k {1 + g_u}$, where *A*_1_ is the initial (year-one) appropriation defined by a funding regime (Supplementary Tables 1–3) and *g*_*u*_ ∈ [0, 1] is the appropriation growth rate in period *t*_*u*_, with *g*_1_ = 0 (Supplementary Table 4). An appropriation *A*_*i*_ funds DAC vintage *i* over the vintage’s operating lifetime, with *A*_*i*_ allocated equally (annualized) over periods $t_{{\mathrm{{\Omega} }}_i}$, given by $\alpha _{i,k} = A_iL^{ - 1}\,\forall k \in {\Omega} _i$ and 0 otherwise. It follows that the appropriation “disbursed” in period *k* (in Fig. 2a) is given by $\hat \alpha _k = \mathop {\sum}\nolimits_{u,v = 1}^k {a_{u,v}}$.

### Energy use, CO_2_ emissions and removal, and cost

Calculation of DAC deployment and of associated energy use, CO_2_ removal, and cost is iterative by DAC vintage and follows four steps broadly: calculation of plant-level totals, calculation of new deployment and retirement, application of technological learning-by-doing, and, after the iteration process is completed, calculation of fleet-aggregated totals and levelized totals (Supplementary Fig. 2).

Electricity use by DAC is given by $\eta _{i,k}^{{\mathrm{dac}}} = \dot E_i^{{\mathrm{dac}}}U{\Delta} \,\forall {\mathrm{k}} \in {\Omega} _{\mathrm{i}}$, where $\dot E^{{\mathrm{dac}}} = E^{{\mathrm{dac}}}R^{{\mathrm{dac}}}8760^{-1}$ is the electricity demand of the DAC process in kWh h^–1^, and $U = \min \{ U^{{\mathrm{dac}}},U^{{\mathrm{elec}}} + U^{{\mathrm{es}}}\} \in [0,8760]$ is the plant uptime (availability) in hours of annual operation and constrained by either the uptime of the DAC process *U*^dac^ or the total electricity resource *U*^elec^ + *U*^es^ (electric grid “elec” plus energy storage “es”). *E*^dac^ includes the compressor load, which compresses CO_2_ to 15 MPa for pipeline injection and is identical across configurations. Electricity is also consumed by heat pumps, $\eta _{i,k}^{{\mathrm{hp}}} = 0.0036r_i^{{\mathrm{hp}}}\beta _i^{-1}{\mathrm{U}}{\Delta} \,\forall {\mathrm{k}} \in {\Omega} _{\mathrm{i}}$, where *r*^hp^ is the is the capacity of installed heat pumps in GJ-thermal (GJt), with $r_i^{{\mathrm{hp}}} = \dot H_i^{{\mathrm{dac}}}\,\forall s \in {\cal{S}}_{{\mathrm{LT}}-{\mathrm{hp}}}$ and 0 otherwise, $\dot H^{{\mathrm{dac}}} = H^{{\mathrm{dac}}}R^{{\mathrm{dac}}}8760^{-1}$ is the low-temperature heat demand in GJ h^–1^, and *β* is the heat pump coefficient of performance in GJt GJe^–1^. It follows that total electricity consumption at the DAC plant is $\eta = \eta ^{{\mathrm{dac}}} + \eta ^{{\mathrm{hp}}}$.

Natural gas is combusted for process heat in scenarios $s \in \{ {\cal{S}}_{{\mathrm{HT}}-{\mathrm{g}}},{\cal{S}}_{{\mathrm{LT}}-{\mathrm{g}}}\}$. For HT-g DAC, gas use is given by $\gamma _{i,k}^{{\mathrm{dac}}} = {\mathrm{LHV}}^{-1}\dot G_i^{{\mathrm{dac}}}U{\Delta} \,\forall {\mathrm{k}} \in {\Omega} _{\mathrm{i}}$, where LHV is the lower heating value of natural gas in GJ t^–1^; for LT-g DAC, $\gamma _{i,k}^{{\mathrm{dac}}} = {\mathrm{HHV}}^{-1}{\mathrm{Eff}}^{{\mathrm{boil}}^{ - 1}}\dot H_i^{{\mathrm{dac}}}U{\Delta} \,\forall {\mathrm{k}} \in {\Omega} _{\mathrm{i}}$, where Eff^boil^ is the boiler efficiency. For all other configurations, *γ*^dac^ = 0. HT-g DAC is defined by a gas requirement $\dot G^{{\mathrm{dac}}} = G^{{\mathrm{dac}}}R^{{\mathrm{dac}}}8760^{-1}$ (GJ h^–1^), whereas LT DAC is defined by the low-temperature heat requirement $\dot H^{{\mathrm{dac}}}$ (GJ h^–1^) that can be supplied via gas combustion or otherwise (e.g., waste heat or heat pumps). Combined cycle gas turbines (CCGT) consume natural gas when serving as the electricity source, given by $\gamma _{i,k}^{{\mathrm{ccgt}}} = \tilde \eta _{i,k}^{{\mathrm{grid}}}q_k{\mathrm{LHV}}^{-1}$, where $\tilde \eta _{i,k}^{{\mathrm{grid}}} = \eta _{i,k}U^{-1}\left( {U^{{\mathrm{elec}}} + U^{{\mathrm{es}}}{\mathrm{Eff}}^{{\mathrm{es}}^{-1}}} \right)$ is power supplied by the grid to the DAC plant and energy storage system, where Eff^es^ is the roundtrip efficiency of energy storage, and *q* is the CCGT net heat rate in GJ kWh^–1^ (where “net” is inclusive of parasitic load for capture and compression to 15 MPa; see Supplementary Tables 9 and 10 for electricity source parameters); otherwise, *γ*^ccgt^ = 0.

Plant CO_2_ emissions derive from electricity generation and production of process heat, given by ${\it{\epsilon }}_{i,k}^{{\mathrm{elec}}} = \tilde \eta _{i,k}^{{\mathrm{grid}}}{\mathrm{CI}}_k^{{\mathrm{elec}}}$ and ${\it{\epsilon }}_{i,k}^{{\mathrm{heat}}} = \gamma _{i,k}^{{\mathrm{dac}}}{\mathrm{LHV}}\,{\mathrm{CI}}_i^{{\mathrm{heat}}}$, respectively, where ${\mathrm{CI}}_k^{{\mathrm{elec}}}$ and ${\mathrm{CI}}_i^{{\mathrm{heat}}}$ are the carbon intensities of electricity generation and heat production, in tCO_2_ kWh^–1^ and tCO_2_ GJ^–1^, respectively (Supplementary Tables 7 and 9). Combustion emissions originate from within the plant boundary (they are direct emissions), while emissions from electricity generation are indirect, but both are attributable in the calculation of net CO_2_ removal. Fugitive leaks of methane from natural gas infrastructure are given by ${\it{\epsilon }}_{i,k}^{{\mathrm{CH}}4} = \lambda \left( {\gamma _{i,k}^{{\mathrm{dac}}} + \gamma _{i,k}^{{\mathrm{ccgt}}}} \right)$, where *λ* ∈ [0, 1] is the fraction of leakage from production, gathering, processing, transmission, and storage (Supplementary Table 11).

CO_2_ captured from the atmosphere is given by $\chi _{i,k}^{{\mathrm{atm}}} = R^{{\mathrm{dac}}}U{\Delta} {\mathrm{/}}8760\,\forall {\mathrm{k}} \in {\Omega} _{\mathrm{i}}$. For scenarios $s \in {\cal{S}}_{{\mathrm{HT}}-{\mathrm{g}}}$, 100% of CO_2_ is captured from the oxy-fuel combustion process, given by $\chi _{i,k}^{{\mathrm{heat}}} = \dot G_i^{{\mathrm{dac}}}\mu {\mathrm{HHV}}^{-1}U{\Delta} \,\forall {\mathrm{k}} \in {\Omega} _{\mathrm{i}}$, where *μ* = 2.744 gCO_2_ gCH_4_^–1^ is the ratio of molecular weights of CO_2_ to CH_4_, HHV is the higher heating value of methane, and assuming 100% conversion of CH_4_ to CO_2_; for all other scenarios, CO_2_ is not captured from heat production, i.e., *χ*^heat^ = 0. CO_2_ is captured when CCGT with post-combustion capture serves as the electricity source, given by $\chi _{i,k}^{{\mathrm{elec}}} = \tilde \eta _{i,k}^{{\mathrm{grid}}}{\mathrm{CC}}_k^{{\mathrm{elec}}}$, where CC^elec^ is the carbon capture factor in tCO_2_-captured per kWh electricity supplied (Supplementary Note 1); otherwise, CC^elec^ = 0. Gross CO_2_ removal from the atmosphere is equivalent to *χ*^atm^, total CO_2_ captured at the DAC plant is given by *χ*^dac^ = *χ*^atm^ + *χ*^heat^, and net CO_2_ removal from the atmosphere, i.e., gross removal less process emissions, is given by $\rho = \chi ^{{\mathrm{atm}}}-{\it{\epsilon }}^{{\mathrm{elec}}}-{\it{\epsilon }}^{{\mathrm{heat}}}$. Capturing CO_2_ from energy generation, though not counted toward gross atmospheric CO_2_ removal, is nevertheless important for maximizing *ρ*.

The total plant cost *c*_*i,k*_ gives the cost of a DAC plant deployed in period *i* during each period of operation *k* ∈ Ω_*i*_. The total cost is comprised of capital and operating costs for the DAC system *c*^dac^ and means of heat production *c*^heat^, energy costs for electricity *c*^elec^ and natural gas *c*^ngas^, and CO_2_ disposal costs *c*^seq^, given by

1 $$c_{i,k} = c_{i,k}^{{\mathrm{dac}}} + c_{i,k}^{{\mathrm{heat}}} + c_{i,k}^{{\mathrm{elec}}} + c_{i,k}^{{\mathrm{ngas}}} + c_{i,k}^{{\mathrm{seq}}}{\,}\forall k \in {\Omega} _i.$$

It follows that the lifetime cost of a plant of vintage *i* is $$\mathop {\sum}\nolimits_k {c_{i,k}}$$. DAC costs are expressed as $$c_{i,k}^{{\mathrm{dac}}} = C_i^{{\mathrm{dac}},{\mathrm{cap}}}R^{{\mathrm{dac}}}\,{\mathrm{CRF}}{\Delta} + C_i^{{\mathrm{dac}},{\mathrm{om}}}R^{{\mathrm{dac}}}{\Delta} \,\forall {\mathrm{k}} \in {\Omega} _{\mathrm{i}}$$, where *C*^dac,cap^ is the plant capital cost in $ tCO_2_^–1^ yr^–1^, *C*^dac,om^ is the plant operating cost in $ tCO_2_^–1^, $${\mathrm{CRF}} = {\mathrm{WACC}}\left( {1 + {\mathrm{WACC}}} \right)^L\left( {\left( {1 + {\mathrm{WACC}}} \right)^L - 1} \right)^{ - 1}$$ is the capital recovery factor in yr^–1^, and WACC is the weighted average cost of capital. Costs are inclusive of CO_2_ compressor costs. Heat production costs are given by $$c_{i,k}^{{\mathrm{heat}}} = C_i^{{\mathrm{boil}},{\mathrm{cap}}}r_{\mathrm{i}}^{{\mathrm{boil}}}{\mathrm{CRF}}{\Delta} + C_i^{{\mathrm{hp}},{\mathrm{cap}}}r_{\mathrm{i}}^{{\mathrm{hp}}}{\mathrm{CRF}}{\Delta} + C^{{\mathrm{hp}},{\mathrm{om}}}r_i^{{\mathrm{hp}}}U{\Delta} \,\forall {\mathrm{k}} \in {\Omega} _{\mathrm{i}}$$, where $$C^{ \cdot ,{\mathrm{cap}}}$$, $$C^{ \cdot ,{\mathrm{om}}}$$, and $$r^{( \cdot )}$$ denote the capital cost, operating cost, and capacity of the boiler “boil” and heat pumps “hp”. Waste heat is taken to have zero cost. Electricity and natural gas costs are given by $$c_{i,k}^{{\mathrm{elec}}} = \eta _{i,k}^{{\mathrm{dac}}}U^{-1}\left( {U^{{\mathrm{elec}}}{\mathrm{MC}}_k^{{\mathrm{elec}}} + U^{{\mathrm{es}}}{\mathrm{MC}}_k^{{\mathrm{es}}}} \right)$$ and $$c_{i,k}^{{\mathrm{ngas}}} = \gamma _{i,k}^{{\mathrm{dac}}}{\mathrm{HHV}}\,{\mathrm{MC}}_k^{{\mathrm{ngas}}}$$, respectively, where MC^(·)^ is commodity marginal cost (electricity “elec”, $ kWh^–1^; energy storage “es”, $ kWh^–1^; natural gas “ngas”, $ GJ^–1^). Natural gas costs are attributed only to gas consumed for heat production; fuel costs for electricity generation are included in MC^elec^. The cost of sequestration is given by $$c_{i,k}^{{\mathrm{seq}}} = \chi _{i,k}^{{\mathrm{dac}}}{\mathrm{MC}}_k^{{\mathrm{seq}}}$$, where MC^seq^ is the marginal cost of CO_2_ transport and sequestration in $ tCO_2_^–1^ (Supplementary Table 12). Sequestration costs are applied only to CO_2_ captured within the plant boundary; the costs of capturing CO_2_ from electricity generation are included in MC^elec^. All costs are set to a 2018$ basis.

In our framework DAC plants are treated as government-mandated expenses and thus bear no capital risk beyond any other highly credible government mandate; hence we set WACC to zero. Supplementary Figure 20 explores the importance of risk-adjusted capital costs, showing sensitivity to variation in WACC over the full span of U.S. long-term Treasury Bills covering the last three decades.

### Deployment

Deployment of DAC plants is tracked by vintage. Plants are constructed, placed into service for their operating lifetime $t_{{\mathrm{{\Omega} }}_i}$, then retired. Deployment and fleet size are tracked via the number of new plants deployed $\pi _i^{{\mathrm{new}}}$, retired $\pi _i^{{\mathrm{ret}}}$, and operating *π*_*i*_. New deployment is constrained either by available funding or the rate at which the DAC industry can scale. Given plant total cost *c*_*i,k*_, the appropriation *a*_*i*_ permits construction of $\mathop {\sum}\nolimits_k {\alpha _i{\mathrm{/}}c_{i,k}}$ new plants in period *t*_*i*_. Industry growth is defined with a maximum initial deployment and growth rate. The initial deployment is a ceiling *n* on the number of plants that can be deployed at the start of the program, i.e., $\pi _1^{{\mathrm{new}}} \le n$ and we set *n* *=* 5. A dynamic diffusion constraint relates the construction of plants in period *i* to the previous period *i* – 1, given by $\pi _i^{{\mathrm{new}}} \le \pi _{i-1}^{{\mathrm{new}}}\left( {1 + p} \right)$, where $p \in \left[ {0,1} \right]$ is the maximum industry growth rate. We set *p* = 0.2, in line with prior use and historical growth of energy technologies^21^. The number of plants deployed in period *t*_*i*_ is therefore given by $\pi _i^{{\mathrm{new}}} = \min \left\{ {\mathop {\sum}\nolimits_k {\alpha _i{\mathrm{/}}c_{i,k}} ,\pi _{i-1}^{{\mathrm{new}}}\left( {1 + p} \right)} \right\}$. Plants are retired at end-of-operating-life, given by $\pi _i^{{\mathrm{ret}}} = \pi _{i - L/{\Delta} }^{{\mathrm{new}}}\,\forall i {\,}> {\,} L{\mathrm{/}}{\Delta}$ and 0 otherwise. Constraints on deployment lead to the logistic (“S”-shaped) growth characteristic of industries that emerge, expand, and saturate in the marketplace. The total number of plants operating in period *t*_*i*_ is the cumulative sum of prior deployments and retirements, i.e., $\pi _i = \mathop {\sum}\nolimits_{u = 1}^i {\pi _u^{{\mathrm{new}}}-\pi _u^{{\mathrm{ret}}}}$.

### Learning

DAC attributes for cost (*C*^dac,cap^, *C*^dac,om^) and energy demand (electricity *E*^dac^, natural gas *G*^dac^, and heat *H*^dac^) improve endogenously through investment and learning, given by

2 $$\phi _i = \phi _1\left( {\frac{{\mathop {\sum}\nolimits_{u,v = 1}^i {\chi _{u,v}^{{\mathrm{atm}}}} }}{{\chi _{1,1}^{{\mathrm{atm}}}}}} \right)^{ - b}$$

3 $$1 - {\mathrm{LR}}^{(\phi )} = 2^{ - b}$$

where *ϕ* represents, independently, each of the five parameters; *ϕ*_1_ is the parameter value in period one; $\chi _{1,1}^{{\mathrm{atm}}}$ is gross atmospheric CO_2_ removal in period one; and LR^(*ϕ*)^ is the learning rate associated with parameter *ϕ*, i.e., the fractional reduction in *ϕ* associated with a doubling of gross removal. Learning effects accrue with each new DAC vintage and are bound by floor estimates on performance and cost. Exogenous learning is applied per forecasts (Supplementary Table 13) for energy supply technologies: CCGT with and without CCS (heat rate, marginal cost, carbon intensity, carbon capture factor), SMRs (marginal cost), lithium-ion battery storage (marginal cost), and heat pumps (capital cost, coefficient of performance).

### Levelized cost of removal and energy use

After the iterative calculations are completed for the modeling period *t*, the marginal levelized cost of net CO_2_ removal (LCOR), in 2018$ tCO_2_^–1^, as well as marginal energy consumption per tCO_2_ net removal EC, in GJ tCO_2_^–1^, are calculated:

4 $${\mathrm{LCOR}}_i = \mathop {\sum}\limits_k {c_{i,k}{\mathrm{/}}\rho _{i,k}}$$

5 $${\mathrm{EC}}_i = \mathop {\sum}\limits_k {{\Xi} _{i,k}{\mathrm{/}}\rho _{i,k}}$$

where ${\mathrm{{\Xi} }} = 0.0036\tilde \eta ^{{\mathrm{grid}}} + {\mathrm{LHV}}\left( {\gamma ^{{\mathrm{dac}}} + \gamma ^{{\mathrm{ccgt}}}} \right)$ is the total energy use in GJ yr^–1^. Marginal values give the lifetime cost and performance by DAC vintage.

### Climate modeling

Two quantities—fleet-aggregated net CO_2_ removal, given by $\hat \rho _k = \mathop {\sum}\nolimits_i {\pi _i^{{\mathrm{new}}}\rho _{i,k}}$, along with fugitive methane emissions attributable to DAC, given by ${{\hat \epsilon }}_k^{{\mathrm{CH}}4} = \mathop {\sum}\nolimits_i {\pi _i^{{\mathrm{new}}}{\it{\epsilon }}_{i,k}^{{\mathrm{CH}}4}}$—are input to two climate models to calculate the impact of removals on net global CO_2_ emissions, atmospheric CO_2_ concentration, and global mean temperature. The impact of removals is quantified relative to baseline futures (for emissions, concentration, and temperature) defined by SSPs^49^; that is, we assume DAC deployment is pursued in concert with mitigation efforts, not in place of them. When calculating impacts beyond 2100, we assume SSP emissions, DAC CO_2_ removals, and fugitive CH_4_ emissions in 2100 remain constant thereafter.

The two climate models, which have been developed independently, simulate the growth rate of atmospheric CO_2_, radiative forcing, and realized global mean warming as a function of time, given an evolution of CO_2_ emissions. The first is a climate-carbon-geochemistry model and has been tested extensively for use in climate mitigation studies^39,55–57^. It contains a carbon cycle model and a one-layer energy balance model. The second is the Minimum Complexity Earth Simulator (MiCES)^40^, which has also been comprehensively tested^58^. It features the coupling of a simpler carbon cycle model and two-layer ocean energy balance model. We include two independent climate models in this study to track variations that stem from model structural difference.

Climate responses to changes in emissions remain an important area of uncertainty in climate science. The two climate models we use match well to past warming. To facilitate comparisons we document how these models compare with MAGICC, which was used to estimate warming for the SSPs. Within the realm of unknowns about the carbon cycle and climate response, our climate models produce CO_2_ concentrations and temperatures in 2100 with mean absolute difference of 14% and 6%, respectively, relative to MAGICC (Supplementary Fig. 21).

### Reporting summary

Further information on research design is available in the Nature Research Reporting Summary linked to this article.

## Supplementary information

Supplementary information

Reporting Summary

## Data Availability

The data that support the plots within this paper and other findings of this study are available from the corresponding author upon reasonable request.

## Code availability

The computer codes that support the analysis within this paper are available from the corresponding author upon reasonable request.
