# Supplementary material for: Emergency deployment of direct air capture as a response to the climate crisis
Source: Nat Commun. 2021 Jan 14;12:368. doi: 10.1038/s41467-020-20437-0 (PMC7809262; doi:10.1038/s41467-020-20437-0)
Supplement: Supplementary file 1 — Supplementary information [file 41467_2020_20437_MOESM1_ESM.pdf]

Supplementary Information

Emergency deployment of direct air capture as a response to the climate crisis

Ryan Hanna[1,2*], Ahmed Abdulla[2,3], Yangyang Xu[4] and David G. Victor[2,5,6,7]

[1] Center for Energy Research, University of California San Diego, La Jolla, CA 92093, USA
[2] Deep Decarbonization Initiative, University of California San Diego, La Jolla, CA 92093, USA
[3] Department of Mechanical and Aerospace Engineering, Carleton University, Ottawa, ON K1S 5B6, Canada
[4] Department of Atmospheric Sciences, Texas A&M University, College Station, TX 77843, USA
[5] School of Global Policy and Strategy, University of California San Diego, La Jolla, CA 92093, USA
[6] Scripps Institution of Oceanography, University of California San Diego, La Jolla, CA 92093, USA
[7] The Brookings Institution, Washington, D.C. 20036
* Corresponding author e-mail address: rehanna@ucsd.edu (R. Hanna)

Supplementary Figures **page #**

Figure S-1	Detailed conceptual schematic of the modeling framework	4
Figure S-2	Calculation process flow for the DAC deployment model	5
Figure S-3	Fuel and CO_2 flows for liquid solvent high-temperature (HT) DAC configurations	6
Figure S-4	Fuel and CO_2 flows for solid sorbent low-temperature (LT) DAC configurations	7
Figure S-5	Net CO_2 removal for individual scenarios by funding regime	21
Figure S-6	New DAC deployment for individual scenarios by funding regime	22
Figure S-7	Size of the operational DAC fleet for individual scenarios by funding regime	23
Figure S-8	Climate benefits of net CO_2 removal assuming SSP5-8.5, marker SSP2, SSP2-4.5, and SSP1-2.6 emission futures	24
Figure S-9	Levelized cost of net CO_2 removal (LCOR) by DAC configuration	25
Figure S-10	Levelized cost of net CO_2 removal (LCOR) by system component: high-temperature DAC	26
Figure S-11	Levelized cost of net CO_2 removal (LCOR) by system component: low-temperature DAC	27
Figure S-12	Process emissions by scenario	28
Figure S-13	Levelized cost of CO_2 capture, process emissions, and levelized cost of net CO_2 removal (LCOR)	29
Figure S-14	Appraisal of scenarios by net CO_2 removal and energy use	30
Figure S-15	Growth in natural gas and electricity use	31
Figure S-16	Net CO_2 removal sensitivity to upscaling, DAC plant, and energy system parameters	32
Figure S-17	Total expenditure sensitivity to upscaling, DAC plant, and energy system parameters	33
Figure S-18	Net CO_2 removal sensitivity to variation in daily hours of renewable power for scenarios with renewables as the electricity supply	34
Figure S-19	Net CO_2 removal sensitivity to variation in daily hours of renewable power for scenarios with renewables plus CCGT as the electricity supply	35
Figure S-20	Net CO_2 removal sensitivity to variation in weighted average cost of capital (WACC)	36
Figure S-21	Comparison of climate model output	37

Supplementary Tables page

Table S-1	Program appropriation in year one for the case of U.S. unilateral funding	8
Table S-2	Program appropriation in year one for the club of democracies (OECD) funding regime	9
Table S-3	Program appropriation in year one for the world cooperation (IBRD) funding regime	10
Table S-4	Projected United States GDP growth	11
Table S-5	DAC configurations considered in this study	12
Table S-6	DAC system parameters and data	13
Table S-7	Heat supply parameters and data	15
Table S-8	Electricity supplies considered in this study	16
Table S-9	Electricity supply parameters and data	17
Table S-10	Energy storage parameters and data	18
Table S-11	Natural gas parameters and data	18
Table S-12	CO_2 disposal parameters and data	18
Table S-13	Exogenous learning-by-doing for energy supplies	19
Table S-14	Effects of delaying deployment of DAC	38

Supplementary Notes page

Note S-1	Carbon intensity and carbon capture factors for combined cycle gas turbines (with or without CCS)	20

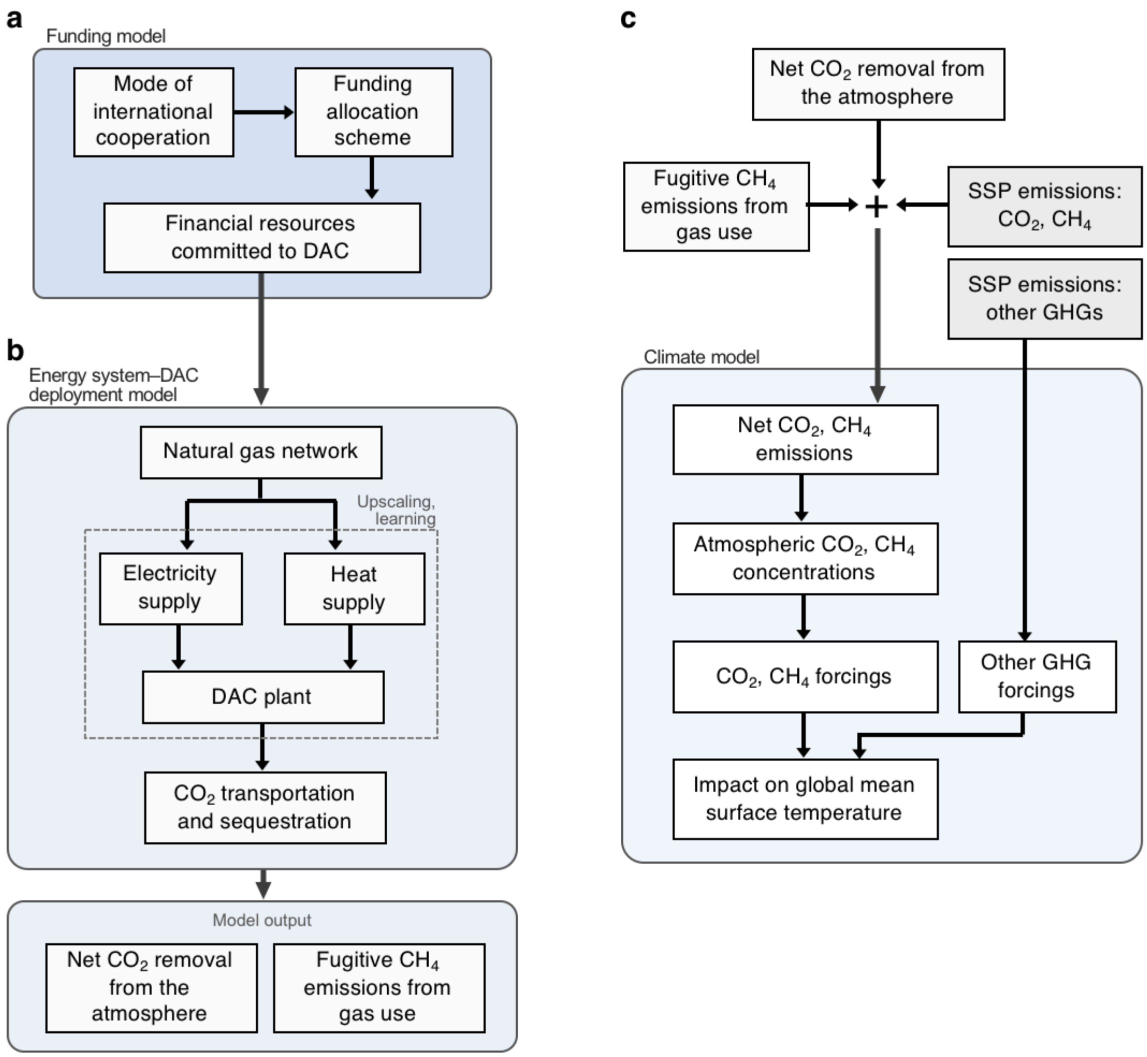

Supplementary Figure 1 | Detailed conceptual schematic of the modeling framework. The model calculates impacts on the climate system due to emergency DAC deployment and comprises three main parts: **a,** a funding model that estimates the financial resources available for DAC deployment; **b,** deployment of a fleet of DAC plants, including requisite supplies of electricity and heat, and upscaling of these DAC-energy system combinations over time; and **c,** the impact of these DAC-energy systems on atmospheric CO_2 concentration and global mean surface temperature, given background emissions which are taken from the shared socioeconomic pathways (SSP).

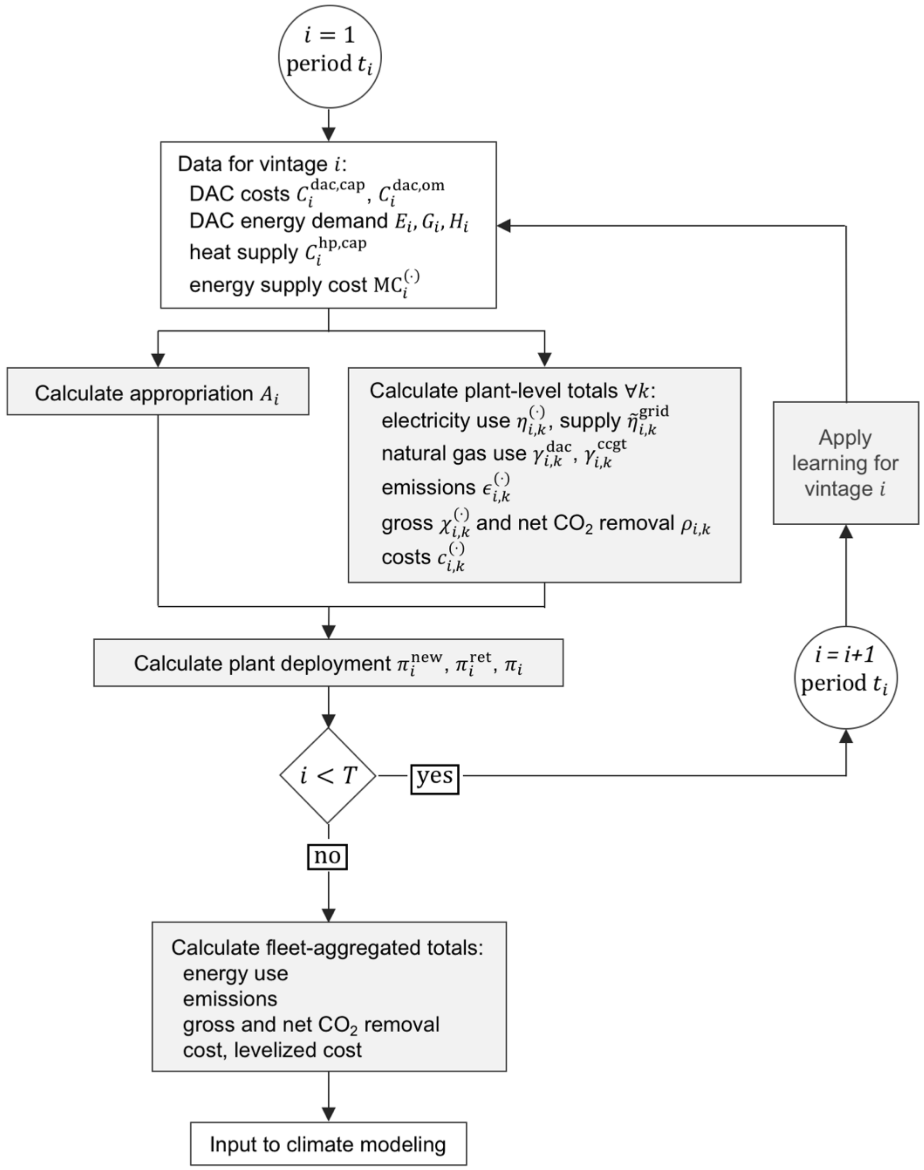

Supplementary Figure 2 | Calculation process flow for the DAC deployment model. Calculation of DAC deployment and associated impacts is iterative over the deployment program $t = \{t_1, \dots, t_T\}$, where T is the number of periods in the program. The calculation has four core components (shaded boxes): calculation of appropriation and plant-level totals; calculation of plant deployment; application of learning; and, once the iterative process finishes, calculation of fleet-aggregated totals. Output is sent to the set of climate models. For reference to notation see the Methods.

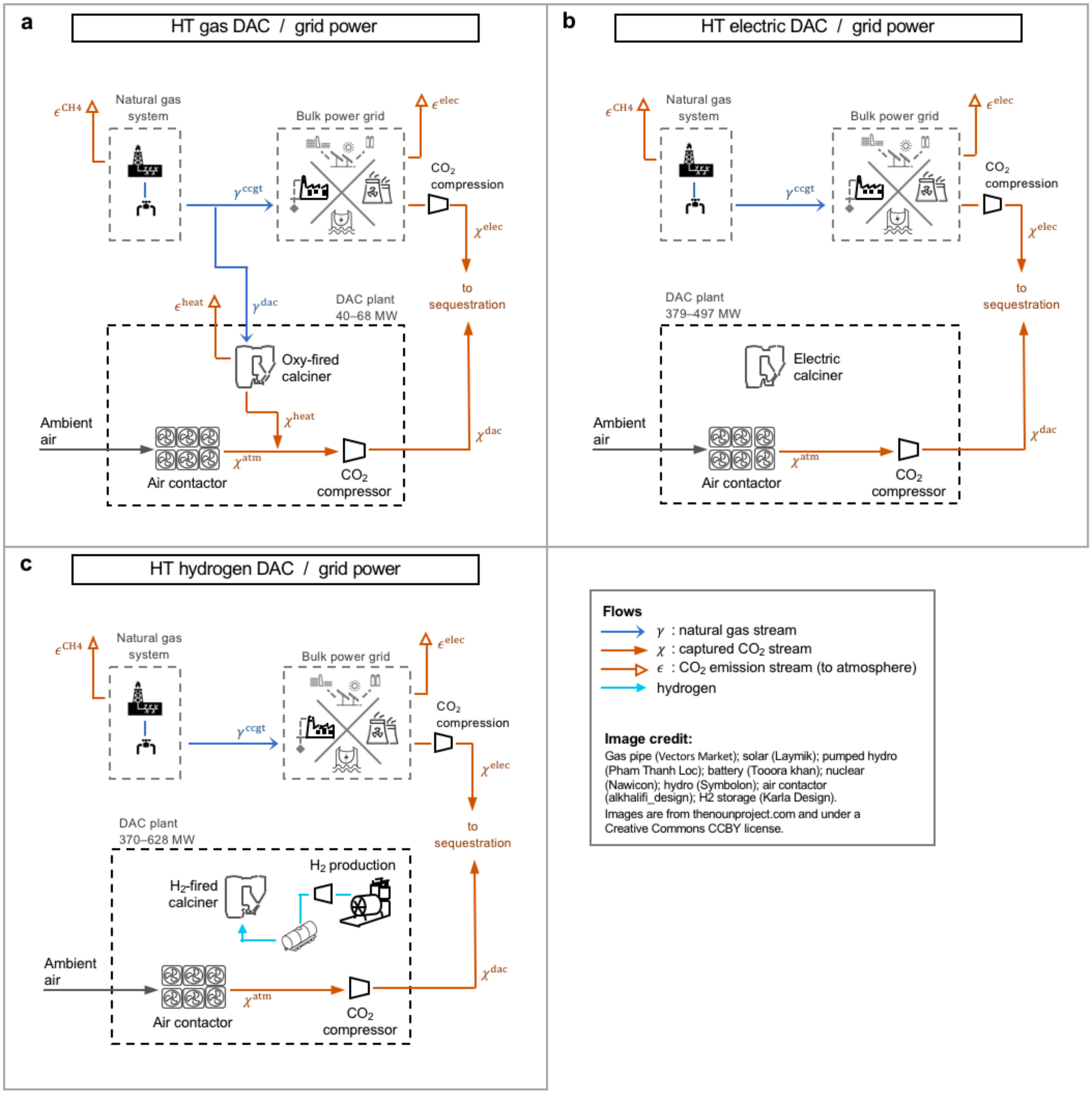

Supplementary Figure 3 | Fuel and CO_2 flows for liquid solvent high-temperature (HT) DAC configurations. Configurations vary in their supply of process heat: **a,** HT DAC with a gas-fired oxy-combustion calciner; **b,** HT DAC with an electric calciner; **c,** HT DAC with a hydrogen-fired calciner, which includes a full production line for hydrogen, including electrolyzer, compressor, and storage. Also shown in each configuration are sources of fuel and electricity, sources of CO_2 emissions and capture, and major sources of energy demand. All configurations are grid-connected. Variation in DAC electric demand stems from process improvement via technological learning. The three major subsystems—DAC plant, electric grid, and gas network—are boxed. For reference to notation see the Methods.

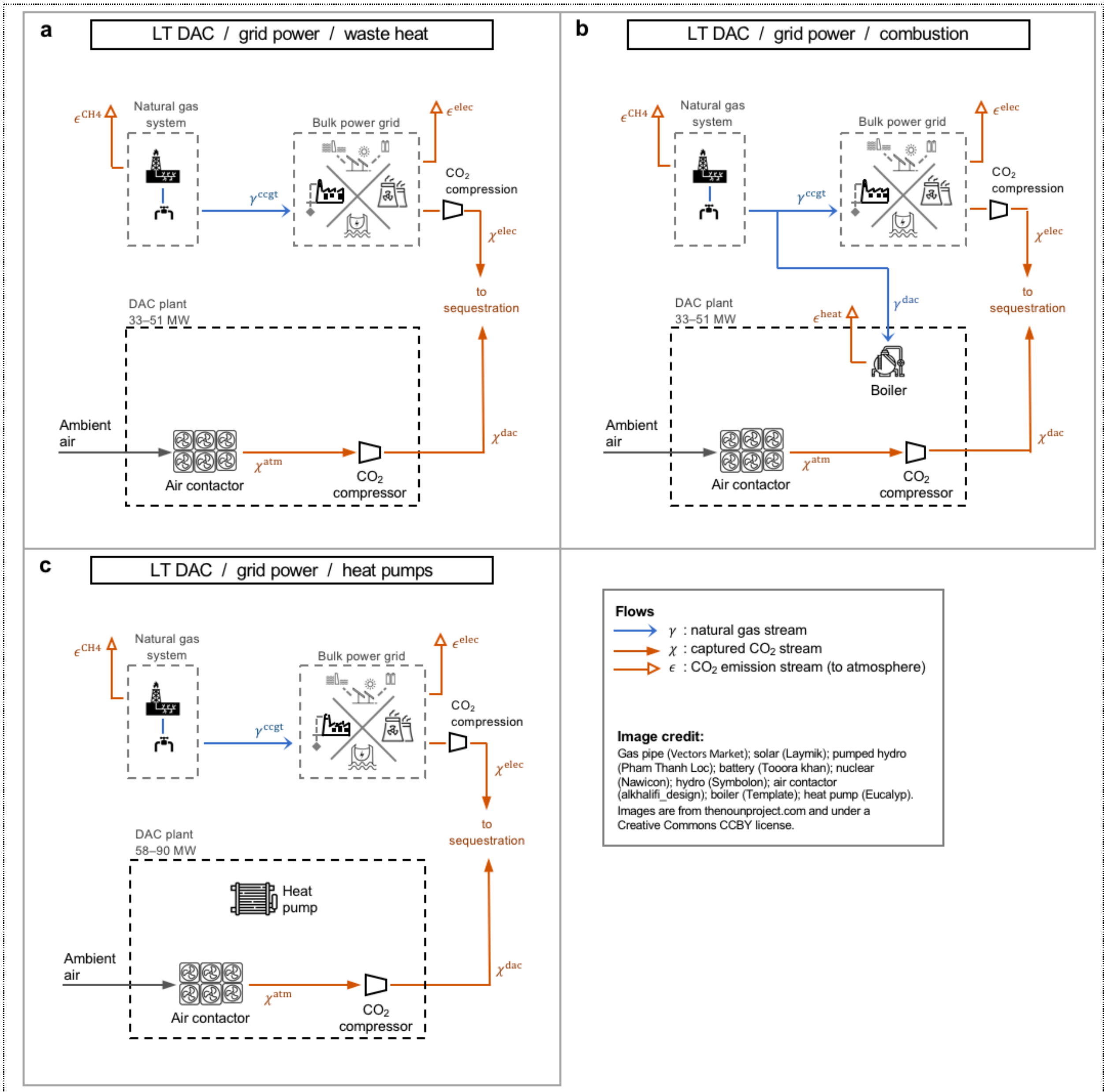

Supplementary Figure 4 | Fuel and CO_2 flows for solid sorbent low-temperature (LT) DAC configurations. Configurations vary in their supply of process heat: **a,** LT DAC using waste; **b,** LT DAC with a gas-fired boiler; **c,** LT DAC with heat pumps. Also shown in each configuration are sources of fuel and electricity, sources of CO_2 emissions and capture, and major sources of energy demand. All configurations are grid-connected. Variation in DAC electric demand stems from process improvement via technological learning. The three major subsystems—DAC plant, electric grid, and gas network—are boxed. For reference to notation see the Methods.

Supplementary Table 1 | Program appropriation in year one for the case of U.S. unilateral funding. The United States is the sole actor, spending 5% of 2018 GDP annually on deployment. This 5% appropriation acts as anchor funding for the collaborative funding regimes in Supplementary Tables 2–3.

	**2018 GDP**	**Allocation**	**Contribution**
	**billion $**	**% of GDP**	**2018$ billion**
**TOTAL APPROPRIATION**			**1025**
UNITED STATES	20,500	5	1025

Supplementary Table 2 | Program appropriation in year one for the club of democracies (OECD) funding regime. OECD member nations work as a coalition, with each allocating a percentage of their GDP to deployment. The U.S. contribution is maintained from the unilateral case, while other countries' allocation is a function of their contribution to the 2017 OECD budget (https://www.oecd.org/about/budget/) relative to the United States' contribution. For example, column 3 shows that Japan contributed 9.4% to the 2017 OECD budget, or 46% (9.4/20.6) of the U.S. share. Thus, Japan contributes 46% of the U.S. anchor funding of 5%, or 2.28%, of their GDP to deployment. And so forth for all nations. Nations are shown in descending order of their 2017 budget contribution.

Nation	2018 GDP	2017 OECD Budget Contribution	Allocation	Contribution
	billion $	% of total	% of GDP	2018$ billion
**TOTAL APPROPRIATION**				**1392**
UNITED STATES	20,500	20.6	5	1025
JAPAN	4970	9.4	2.28	113.4
GERMANY	4000	7.4	1.80	71.8
UNITED KINGDOM	2830	5.5	1.33	37.8
FRANCE	2780	5.4	1.31	36.4
ITALY	2070	4.1	1.00	20.6
CANADA	1710	3.6	0.87	14.9
AUSTRALIA	1430	3.1	0.75	10.8
KOREA	1620	3.1	0.75	12.2
SPAIN	1430	3	0.73	10.4
MEXICO	1220	2.8	0.68	8.3
NETHERLANDS	910	2.2	0.53	4.9
SWITZERLAND	710	2.1	0.51	3.6
TURKEY	770	2	0.49	3.7
BELGIUM	530	1.6	0.39	2.1
NORWAY	430	1.6	0.39	1.7
SWEDEN	550	1.6	0.39	2.1
AUSTRIA	460	1.5	0.36	1.7
POLAND	590	1.5	0.36	2.1
CHILE	300	1.4	0.34	1.0
ESTONIA	30	1.4	0.34	0.1
ISRAEL	400	1.4	0.34	1.4
LATVIA	30	1.4	0.34	0.1
SLOVENIA	50	1.4	0.34	0.2
DENMARK	350	1.3	0.32	1.1
FINLAND	280	1.2	0.29	0.8
CZECH REPUBLIC	240	1.1	0.27	0.6
GREECE	220	1.1	0.27	0.6
IRELAND	380	1.1	0.27	1.0
NEW ZEALAND	210	1.1	0.27	0.6
PORTUGAL	240	1.1	0.27	0.6
HUNGARY	160	1	0.24	0.4
SLOVAK REPUBLIC	110	0.8	0.19	0.2
LUXEMBOURG	70	0.6	0.15	0.1
ICELAND	30	0.5	0.12	0.0

Supplementary Table 3 | Program appropriation in year one for the world cooperation (IBRD) funding regime. IBRD member nations work as a coalition, with each allocating a percentage of their GDP to deployment. The U.S. contribution is maintained from the unilateral case, while other countries' allocation is a function of their 2018 IBRD subscription holdings (https://finances.worldbank.org/) relative to the United States holding. For example, Japan holds 8.31% of subscriptions, or 8.31/16.57 = 50% of the U.S. share of the IBRD subscriptions. Thus, Japan contributes 50% of the U.S. anchor funding of 5%, or 2.5%, of their GDP to deployment. Nations shown, in descending order of their 2018 subscription holding, are inclusive of >99.5% of the total appropriation; the remainder contribute <0.5% and are omitted for brevity.

	**2018 GDP**	**IBRD Subscriptions**	**Allocation**	**Contribution**
	**billion $**	**% of total**	**% of GDP**	**$2018 billion**
**TOTAL APPROPRIATION**				**1612**
UNITED STATES	20,500	16.57	5	1025
JAPAN	4971	8.31	2.51	124.7
CHINA	13608	4.59	1.39	188.5
GERMANY	3997	4.16	1.26	50.2
FRANCE	2778	3.89	1.17	32.6
UNITED KINGDOM	2825	3.89	1.17	33.2
INDIA	2726	3.14	0.95	25.8
CANADA	1709	3.04	0.92	15.7
RUSSIAN FEDERATION	1658	2.87	0.87	14.4
SAUDI ARABIA	782	2.87	0.87	6.8
ITALY	2074	2.73	0.82	17.1
BRAZIL	1869	2.31	0.70	13.0
NETHERLANDS	913	1.97	0.59	5.4
SPAIN	1430	1.90	0.57	8.2
MEXICO	1224	1.73	0.52	6.4
KOREA, REPUBLIC OF	1619	1.62	0.49	7.9
BELGIUM	532	1.61	0.49	2.6
IRAN, ISLAMIC REPUBLIC OF	454	1.51	0.46	2.1
SWITZERLAND	710	1.49	0.45	3.2
AUSTRALIA	1432	1.36	0.41	5.9
ARGENTINA	518	1.14	0.34	1.8
TURKEY	767	1.10	0.33	2.5
INDONESIA	1042	0.99	0.30	3.1
SWEDEN	551	0.85	0.26	1.4
KUWAIT	142	0.84	0.25	0.4
DENMARK	351	0.77	0.23	0.8
SOUTH AFRICA	366	0.77	0.23	0.9
POLAND	586	0.74	0.22	1.3
NIGERIA	397	0.7	0.21	0.8
AUSTRIA	456	0.63	0.19	0.9
UKRAINE	131	0.6	0.18	0.2
NORWAY	435	0.58	0.18	0.8
ALGERIA	181	0.51	0.15	0.3
PAKISTAN	313	0.51	0.15	0.5
…	…	…	…	…

Supplementary Table 4 | Projected United States GDP growth. Growth rates are the 5-year average growth rates across the three growth-central Shared Socioeconomic Pathways (SSP1, SSP2, SSP4) and three GDP models in the SSP database (IIASA GDP, OECD Env-Growth, PIK GDP-32)[1-3].

**Year**	**Growth (% yr^{-1})**
2025	2.4
2030	2.1
2035	1.9
2040	1.8
2045	1.6
2050	1.4
2055	1.3
2060	1.3
2065	1.2
2070	1.1
2075	1.0
2080	1.0
2085	0.9
2090	0.8
2095	0.8
2100	0.7

Supplementary Table 5 | DAC configurations considered in this study. Configurations consist of a DAC process collocated with and bound to a supply of heat. Heat supplies can be particular to a DAC process—either because the integrated system is co-designed, as with high-temperature (HT) DAC, or because requirements for heat quality allow natural pairings with low-grade or waste heat, as with low-temperature (LT) DAC.

<table>
<tr><th>Configuration No.</th><th>DAC Type</th><th>Heat Supply</th><th>Code</th><th>Reference</th></tr>
<tr><td>1</td><td rowspan="3">LT</td><td>Natural gas combustion/boiler</td><td>LT-g</td><td rowspan="3">NAS 2019 (ref. 4)</td></tr>
<tr><td>2</td><td>Waste heat</td><td>LT-w</td></tr>
<tr><td>3</td><td>Heat pumps</td><td>LT-hp</td></tr>
<tr><td rowspan="2">4</td><td rowspan="2">HT gas</td><td rowspan="2">Oxy-fired kiln
(natural gas combustion with CO_2 capture)</td><td rowspan="2">HT-g</td><td>NAS 2019 (ref. 4)</td></tr>
<tr><td>Keith et al. 2018 (ref. 5)</td></tr>
<tr><td>5</td><td>HT electric</td><td>Electric kiln</td><td>HT-e</td><td rowspan="2">NAS 2019 (ref. 4)</td></tr>
<tr><td>6</td><td>HT hydrogen</td><td>Hydrogen-fired kiln
(hydrogen combustion)</td><td>HT-h</td></tr>
</table>

Supplementary Table 6 | DAC system parameters and data. We model six unique DAC plant configurations (Supplementary Table 5), where a configuration consists of a DAC type and collocated supply of heat. Two estimates, from academia[4] and industry[5] have been reported for the HT gas system. Costs are scaled to a 2018 USD cost basis per the U.S. Bureau of Labor Statistics consumer price index.

**Parameter**	**Variable**	**Units**	**HT gas[a]**	**HT gas[b]**	**HT electric**	**HT hydrogen**	**LT waste heat**	**LT combustion**	**LT heat pump**
Source of data	-	-	NAS 2019 (ref. 4)	Keith et al. 2018 (ref. 5)	NAS 2019 (ref. 4)	NAS 2019 (ref. 4)	NAS 2019 (ref. 4)	NAS 2019 (ref. 4)	NAS 2019 (ref. 4)
Uptime (availability)	U^{dac}	h/yr	7890	7890	7890	7890	7890	7890	7890
Nameplate capacity	R^{dac}	tCO_2/yr	1 000 000	1 000 000	1 000 000	1 000 000	1 000 000	1 000 000	1 000 000
Air contactor capture efficiency	f^{ac}	-	0.75	0.75	0.75	0.75	0.75	0.75	0.75
CO_2 outlet pressure	-	MPa	15	15	15	15	15	15	15
Lifetime	L	yr	25	25	25	25	25	25	25
Capital cost (year 1)	$\overline{C}^{dac,cap}$	\$/tCO_2/yr	1334	1053	769	2112	2170	2170	2170
Capital cost (floor)	$\underline{C}^{dac,cap}$	\$/tCO_2/yr	722	729	592	1120	812	812	812
O&M cost (year 1)	$\overline{C}^{dac,om}$	\$/tCO_2	59.3	38.2	37.3	89.7	23.3	23.3	23.3
O&M cost (floor)	$\underline{C}^{dac,om}$	\$/tCO_2	33.4	27.3	28.3	48.9	11.9	11.9	11.9
Electricity demand (year 1)	$\overline{E}^{dac}$	kWh/tCO_2	594	366	4358	5497	444	444	444
Electricity demand (floor)	$\underline{E}^{dac}$	kWh/tCO_2	350	366	3322	3244	286	286	286
Natural gas demand (year 1)	$\overline{G}^{dac}$	GJ/tCO_2	12.2	5.3	–	–	–	–	–
Natural gas demand (floor)	$\underline{G}^{dac}$	GJ/tCO_2	5.3	5.3	–	–	–	–	–
Heat demand (year 1)	$\overline{H}^{dac}$	GJ/tCO_2	–	–	–	–	4.8	4.8	4.8
Heat demand (floor)	$\underline{H}^{dac}$	GJ/tCO_2	–	–	–	–	3.4	3.4	3.4

Learning rate for costs	LR	%	10	10	10	10	10	10	10
Learning rate for energy demand	LR	%	2	2	2	2	2	2	2

[a] Variation in initial and floor data stems from design differences. The 2019 National Academies report[4] considers a system with and without the major process improvements in Keith et al. 2018 (ref. 5), such as for contactor design, heat recovery via integrating sub-processes, which affects both cost and energy use. The improved NAS system mirrors the improved Keith et al. 2018 design and therefore its costs and energy use match Keith et al. 2018, while the base case system omits those design improvements, resulting in higher cost and energy demand.
[b] Initial and floor data derive from the first-of-a-kind and Nth-of-a-kind plant financials specified in Keith et al. 2018 (ref. 5). Capex and opex therefore vary but not energy demand.

Supplementary Table 7 | Heat supply parameters and data. Heat sources are proximate to and paired with specific DAC types, given that the requirement for heat quality varies by DAC type. HT DAC is paired with oxy-fired combustion (HT gas), an electric kiln (HT electric), or a hydrogen-fired kiln (HT hydrogen). LT DAC can be paired with a natural gas-fired boiler (LT combustion), waste heat (LT waste heat), or heat pumps (LT heat pump). Costs are scaled to a 2018 USD cost basis per the U.S. Bureau of Labor Statistics consumer price index.

**Parameter**	**Variable**	**Units**	**HT gas** (ref. 4)	**HT gas** (ref. 5)	**HT electric**	**HT hydrogen**	**LT waste heat**	**LT combustion**	**LT heat pump**
Carbon intensity of heat supply	$\mathrm{CI}^{\mathrm{heat}}$	gCO_2/MJ	0	0	varies[d]	varies[d]	0	53.7	varies[d]
Waste heat marginal cost	-	\$/kWht	–	–	–	–	0	–	–
Low-temperature heat boiler[a]									
Boiler capital cost	$C^{\mathrm{boil,cap}}$	\$/kWt	–	–	–	–	–	174.3	–
Boiler efficiency	$\mathrm{Eff}^{\mathrm{boil}}$	-	–	–	–	–	–	0.92	–
Boiler lifetime	L^{boil}	yr	–	–	–	–	–	25	–
Heat pumps (year 1)[b,c]									
Heat pump capital cost	$\overline{C}^{\mathrm{hp,cap}}$	\$/kWt	–	–	–	–	–	–	282
Heat pump fixed O&M cost	$C^{\mathrm{hp,fom}}$	\$/kWt/yr	–	–	–	–	–	–	2.7
Heat pump variable O&M cost	$C^{\mathrm{hp,vom}}$	\$/kWht	–	–	–	–	–	–	0.0024
Heat pump coefficient of performance	$\overline{\beta}$	kWt/kWe	–	–	–	–	–	–	3.9
Heat pump lifetime	L^{hp}	yr	–	–	–	–	–	–	25

[a] NREL, 2014 (ref. 6)
[b] NREL, 2017 (ref. 7)
[c] See Supplementary Table 13 for exogenous variation over the model horizon.
[d] Varies depending on the source of electricity.

Supplementary Table 8 | Electricity supplies considered in this study. We model six different electricity sources (see Supplementary Tables 9–10 for data) and combine them to form 14 unique electricity supplies. Selection of electricity sources is intended to capture power grids that exist today (CCGT, hydropower-heavy grids), that may plausibly exist given current trends toward decarbonization (high levels of renewable curtailment, combinations of CCGT, renewables, and energy storage), and that do not exist today but represent a future deeply decarbonized electric power system (CCGT with CCS, SMR). Hybrid supplies (No. 6–14) pair energy storage and/or CCGT with curtailed renewables to increase DAC plant utilization (uptime).

**No.**	**Resources**	**Code**	**Description**
1	Renewables	R	Curtailed renewables (7 h day^{-1}); envisions a solar midday peak in a solar-heavy grid
2	Hydropower	Rs	Hydroelectric power-dominant power grid
3	CCGT	C	Combined cycle gas turbine
4	CCGT-CCS	Cc	Combined cycle gas turbine with CO_2 capture and sequestration
5	SMRs	S	Small modular nuclear reactors
6	Renewables, storage	Rs	Curtailed renewables (7 h day^{-1}) firmed with Li-ion battery energy storage (4 h day^{-1})
7			Curtailed renewables firmed with Li-ion battery energy storage (7 h)
8			Curtailed renewables firmed with pumped hydro energy storage (4 h)
9			Curtailed renewables firmed with pumped hydro energy storage (7 h)
10	Renewables, CCGT	R.C	Curtailed renewables and CCGT otherwise
11	Renewables, storage, CCGT	Rs.C	Curtailed renewables (7 h day^{-1}) firmed with Li-ion battery energy storage (4 h day^{-1}) and CCGT otherwise
12			Curtailed renewables firmed with Li-ion battery energy storage (7 h) and CCGT otherwise
13			Curtailed renewables firmed with pumped hydro energy storage (4 h) and CCGT otherwise
14			Curtailed renewables firmed with pumped hydro energy storage (7 h) and CCGT otherwise

Supplementary Table 9 | Electricity supply parameters and data. Electricity sources—six unique in total—represent electric power grids dominated by the representative power source: curtailed renewables, hydropower, CCGT with CCS, CCGT, and SMR. Electricity supplies are not modeled as proximate to the DAC plant; rather, DAC plants draw power from a bulk grid (defined by a representative marginal generator) and not from a specific power plant. We model two types of hybrid electricity supply. First, two types of energy storage (Li-ion and pumped hydro; Supplementary Table 10) are combined with curtailed renewables to increase the availability of the resource. Second, CCGT is combined with renewables and storage to further increase availability to 24 h day^{-1}. Abbreviations: CCGT (combined cycle gas turbine), -CCS (with carbon capture and sequestration), SMR (small modular nuclear reactors).

**Parameter**	**Variable**	**Units**	**Curtailed renewables**	**Hydro-power**	**CCGT-CCS**[f]	**CCGT**[f]	**SMR**
Uptime (availability)	$\mathrm{U}^{\mathrm{elec}}$	h/day	7	24	24	24	24
Carbon intensity (year 1)[a]	$\overline{\mathrm{CI}}^{\mathrm{elec}}$	gCO_2/kWh	45[c]	95[d]	44[g]	357[g]	13.2[h]
Carbon capture (year 1)[a]	$\overline{\mathrm{CC}}^{\mathrm{elec}}$	gCO_2/kWh	–	–	374[g]	0	–
Net heat rate (year 1)[a,b]	$\bar{q}$	btu/kWh	–	–	7972	6797	–
CO_2 capture fraction	f^{pcc}	-	–	–	0.9	–	–
Marginal cost (year 1)[a]	$\overline{\mathrm{MC}}^{\mathrm{elec}}$	\$/MWh	0	10.3[e]	72.1	45.8	128[i]

[a] See Supplementary Table 13 for exogenous variation over the model horizon.
[b] "Net", i.e. inclusive of parasitic load from the CO_2 post-combustion capture unit and compression to 15 MPa.
[c] Hsu et al., 2012 (ref. 8)
[d] EIA (https://www.eia.gov/electricity/data/emissions/)
[e] EIA (https://www.eia.gov/electricity/annual/html/epa_08_04.html)
[f] Rubin et al., 2015 (ref. 9)
[g] See Supplementary Note 1 for derivation.
[h] Carless et al., 2016 (ref. 10)
[i] National Nuclear Laboratory, 2014 (ref. 11)

Supplementary Table 10 | Energy storage parameters and data. Two types of energy storage (Lithium-ion and pumped hydro) are used as complements to curtailed renewables to increase the availability of the renewable resource. Storage is assumed to charge solely from curtailed renewables and thus the carbon intensity of delivered power is zero. Abbreviations: BESS (battery energy storage system; represented by Li-ion chemistry), PHES (pumped hydro energy storage).

Parameter	Variable	Units	BESS	PHES
Uptime (availability)	U^{es}	h/day	varies[b]	varies[b]
Carbon intensity	CI^{es}	gCO_2/kWh	0	0
Roundtrip efficiency	Eff^{es}	-	0.86[c]	0.76[c]
Marginal cost (year 1)[a]	$\overline{MC}^{es}$	$/MWh	180[c]	107[d]

[a] See Supplementary Table 13 for exogenous variation over the model horizon.
[b] Varies per scenario setup; see Supplementary Table 8.
[c] Schmidt et al., 2019 (ref. 12)
[d] Victor et al., 2019 (ref. 13)

Supplementary Table 11 | Natural gas parameters and data.

Parameter	Units	Value	Note/Justification
Higher heating value	GJ/t	55.5	assumed 100% methane
Lower heating value	GJ/t	50	assumed 100% methane
Leakage fraction[a]	%	0.32	Current best practice[b]; OGCI[c]
Price	$/GJ	3.86	market price and forecasts

[a] From production, gathering, processing, and transmission and storage—the sources of the majority of leakage.
[b] Although the impact of fugitive methane emissions is small in this analysis, the problem is serious[14–16], and we expect that a crisis response leading to massive deployment of DAC would strictly enforce best practices for producing and transporting methane that might be needed to power the technology.
[c] https://oilandgasclimateinitiative.com/oil-and-gas-climate-initiative-sets-first-collective-methane-target-for-member-companies/

Supplementary Table 12 | CO_2 disposal parameters and data.

Parameter	Units	Value	Note/Justification
Marginal cost of CO_2 transport and sequestration	$/tCO_2$	10	Rubin et al., 2015 (ref. 9)

Supplementary Table 13 | Exogenous learning-by-doing for energy supplies. While DAC learning is treated endogenously, learning for energy supplies is prescribed exogenously following recent studies and forecasts. Abbreviations: CCGT (combined cycle gas turbine), -CCS (with carbon capture and sequestration), SMR (small modular nuclear reactors), BESS (battery energy storage system).

**Parameter**	**Units**	**2025**	**2030**	**2035**	**2040**	**2045**	**2050**	**2055**	**2060**	**2065**	**2070**	**2075**	**2080**	**2085**	**2090**	**2095**	**2100**
CCGT net heat rate[a]	btu/kWh	6797	6729	6661	6593	6525	6457	6389	6321	6253	6185	6117	6117	6117	6117	6117	6117
CCGT marginal cost[a]	2018$ MWh^{-1}	46	46	45	45	45	44	44	44	44	43	43	43	43	43	43	43
CCGT carbon intensity[a]	gCO_2/kWh	357	353	350	346	343	339	336	332	329	325	321	321	321	321	321	321
CCGT-CCS net heat rate[a]	Btu/kWh	7972	7892	7813	7733	7653	7573	7494	7414	7334	7255	7175	7175	7175	7175	7175	7175
CCGT-CCS marginal cost[a]	2018$ MWh^{-1}	72	71	70	69	68	67	66	65	64	63	62	62	62	62	62	62
CCGT-CCS carbon intensity[a]	gCO_2/kWh	44	43.6	43.2	42.7	42.3	41.9	41.5	41.1	40.7	40.2	39.8	39.8	39.8	39.8	39.8	39.8
CCGT-CCS carbon capture factor[a]	gCO_2/kWh	374	371	367	363	359	356	352	348	344	341	337	337	337	337	337	337
SMR marginal cost[b]	2018$ MWh^{-1}	128	126	124	123	121	119	117	115	114	112	110	110	110	110	110	110
BESS marginal cost[c]	2018$ MWh^{-1}	180	126	101	92	87	82	82	82	82	82	82	82	82	82	82	82
Heat pump capital cost[d]	2018$/kWt	282	254	245	236	227	218	218	218	218	218	218	218	218	218	218	218
Heat pump coefficient of performance[d]	kWt/kWe	3.9	4.0	4.1	4.1	4.2	4.2	4.2	4.2	4.2	4.2	4.2	4.2	4.2	4.2	4.2	4.2

[a] Rubin et al., 2015 (ref. 9)
[b] National Nuclear Laboratory, 2014 (ref. 11)
[c] Schmidt et al, 2019 (ref. 12)
[d] NREL, 2017 (ref. 7)

Supplementary Note 1
Carbon intensity and carbon capture factors for combined cycle gas turbines (with or without CCS)

Here we present our model for CO_2 emissions and CO_2 capture at a combined cycle gas turbine (CCGT) power plant and derive the carbon intensity of electricity generation $\mathrm{CI}^{\mathrm{elec}}$ and the carbon capture factor from electricity generation $\mathrm{CC}^{\mathrm{elec}}$. Both have units of gCO_2 per kWh output.

The power plant supplies electricity $\tilde{\eta}^{\mathrm{grid}}$ and combusts natural gas γ^{ccgt} to do so, which produces CO_2 emissions ϵ^{elec}, a fraction of which may be sequestered χ^{elec}. The carbon intensity and carbon capture factor are given by

$$\mathrm{CI}^{\mathrm{elec}} = \epsilon^{\mathrm{elec}} / \tilde{\eta}^{\mathrm{grid}} \qquad \text{(S1.1)}$$

$$\mathrm{CC}^{\mathrm{elec}} = \chi^{\mathrm{elec}} / \tilde{\eta}^{\mathrm{grid}} \, , \qquad \text{(S1.2)}$$

where ϵ^{elec} is plant emissions in gCO_2, χ^{elec} is CO_2 captured in gCO_2, and $\tilde{\eta}^{\mathrm{grid}}$ is electricity supplied by CCGT in kWh. Emission streams are defined by the carbon balance through the plant and assuming 100% CH_4 and 100% conversion of CH_4 to CO_2. CO_2 emitted is given by $\epsilon^{\mathrm{elec}} = (1 - f^{\mathrm{pcc}})\mu\tilde{\eta}^{\mathrm{grid}} q \mathrm{HHV}^{-1}$, while CO_2 captured is given by $\chi^{\mathrm{elec}} = f^{\mathrm{pcc}}\mu\tilde{\eta}^{\mathrm{grid}} q \mathrm{HHV}^{-1}$, where $f^{\mathrm{pcc}} \in [0,1]$ is the post-combustion capture fraction, μ = 2.744 gCO_2 gCH_4^{-1} is the ratio of molecular weights of CO_2 to CH_4, HHV = 55.5 GJ tCH_4^{-1} is the higher heating value of methane, and q is the plant net heat rate on an HHV basis, where net means inclusive of parasitic load from the capture unit and CO_2 compressor. Parasitic load includes 329 kWh per tCO_2 sequestered for the post-combustion capture unit[9] and 133 kWh per tCO_2 sequestered for the compressor[4]. Note that $\epsilon^{\mathrm{elec}} + \chi^{\mathrm{elec}}$ = 1.

The carbon intensity and carbon capture factor can then be rewritten as

$$\mathrm{CI}_k^{\mathrm{elec}} = (1 - f^{\mathrm{pcc}})\mu q_k \mathrm{HHV}^{-1} \, , \qquad \text{(S1.3)}$$

$$\mathrm{CC}_k^{\mathrm{elec}} = f^{\mathrm{pcc}}\mu q_k \mathrm{HHV}^{-1} \, . \qquad \text{(S1.4)}$$

Following ref. 9, a CCGT (without CCS) in year one is defined by f^{pcc} = 0 and q_1 = 6797 btu kWh^{-1} (50.2% efficient, HHV basis), while a CCGT with CCS is defined by f^{pcc} = 0.9 and q_1 = 7972 btu kWh^{-1} (42.8% net efficiency, HHV basis). The lower efficiency is due to electricity demand from CO_2 capture and compression.

It follows that $\mathrm{CI}_1^{\mathrm{elec}}$ = 355 gCO_2 kWh^{-1} and $\mathrm{CC}_1^{\mathrm{elec}}$ = 0 for the CCGT plant, while $\mathrm{CI}_1^{\mathrm{elec}}$ = 41.6 gCO_2 kWh^{-1} and $\mathrm{CC}_1^{\mathrm{elec}}$ = 374 gCO_2 kWh^{-1} for the CCGT-CCS plant. We add 2.4 gCO_2 kWh^{-1} to both, following ref. 17, to account for life-cycle emissions from construction, decommissioning, and ammonia production. We account for upstream fugitive methane emissions separately via the parameter ϵ^{CH4}. Supplementary Table 13 reports $\mathrm{CI}^{\mathrm{elec}}$ and $\mathrm{CC}^{\mathrm{elec}}$ over the model horizon, including exogenous learning improvement.

Supplementary Results

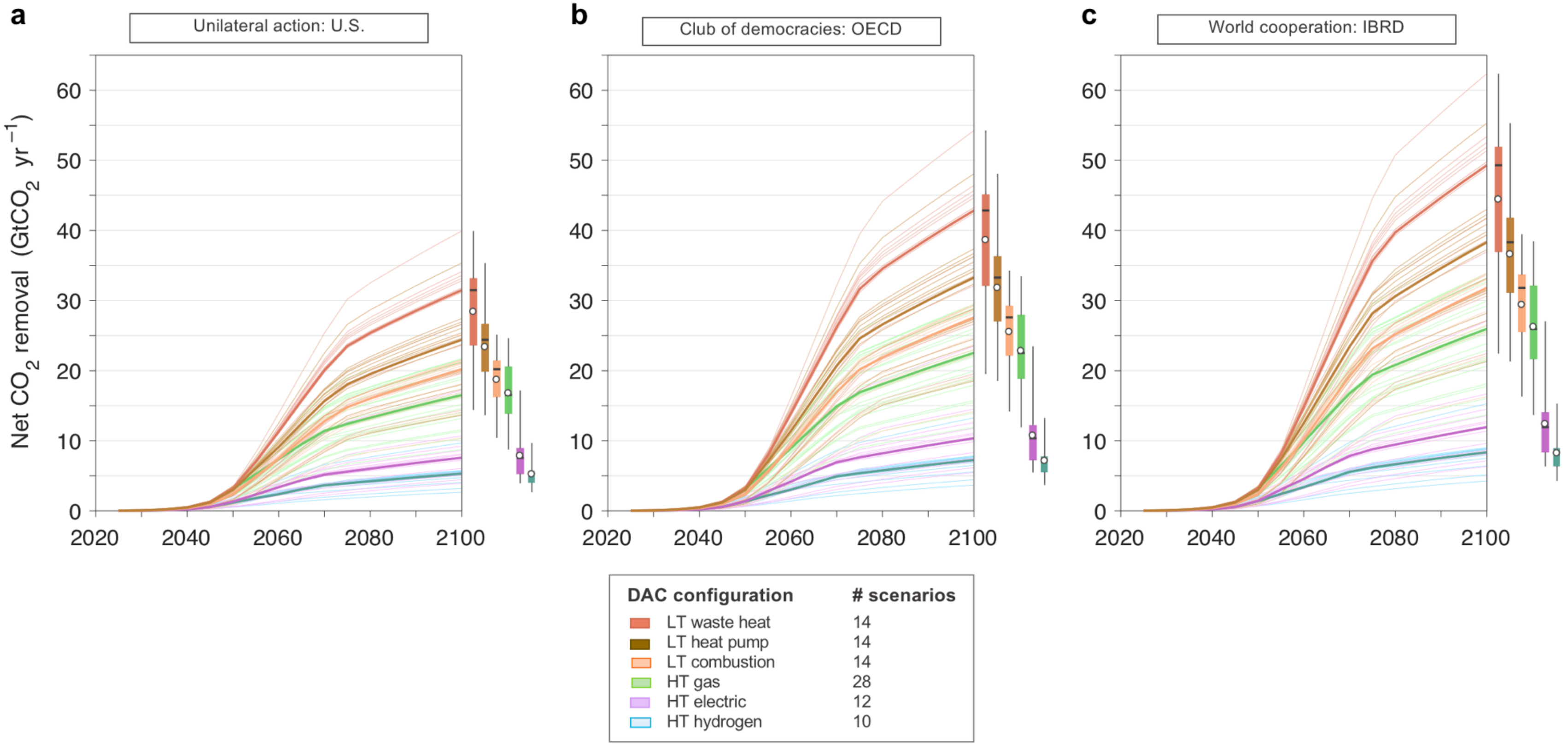

Supplementary Figure 5 | Net CO_2 removal for individual scenarios by funding regime. **a,** U.S. unilateral funding. **b,** funding by the club of advanced democracies (OECD members). **c,** world funding (IBRD members). Scenarios with CO_2 removals >0 are shown for the six DAC configurations, which are distinguished by color. Thick lines denote median scenarios. Boxplots show removal in 2100: center line, median; dot, mean; box limits, interquartile range; whiskers, range.

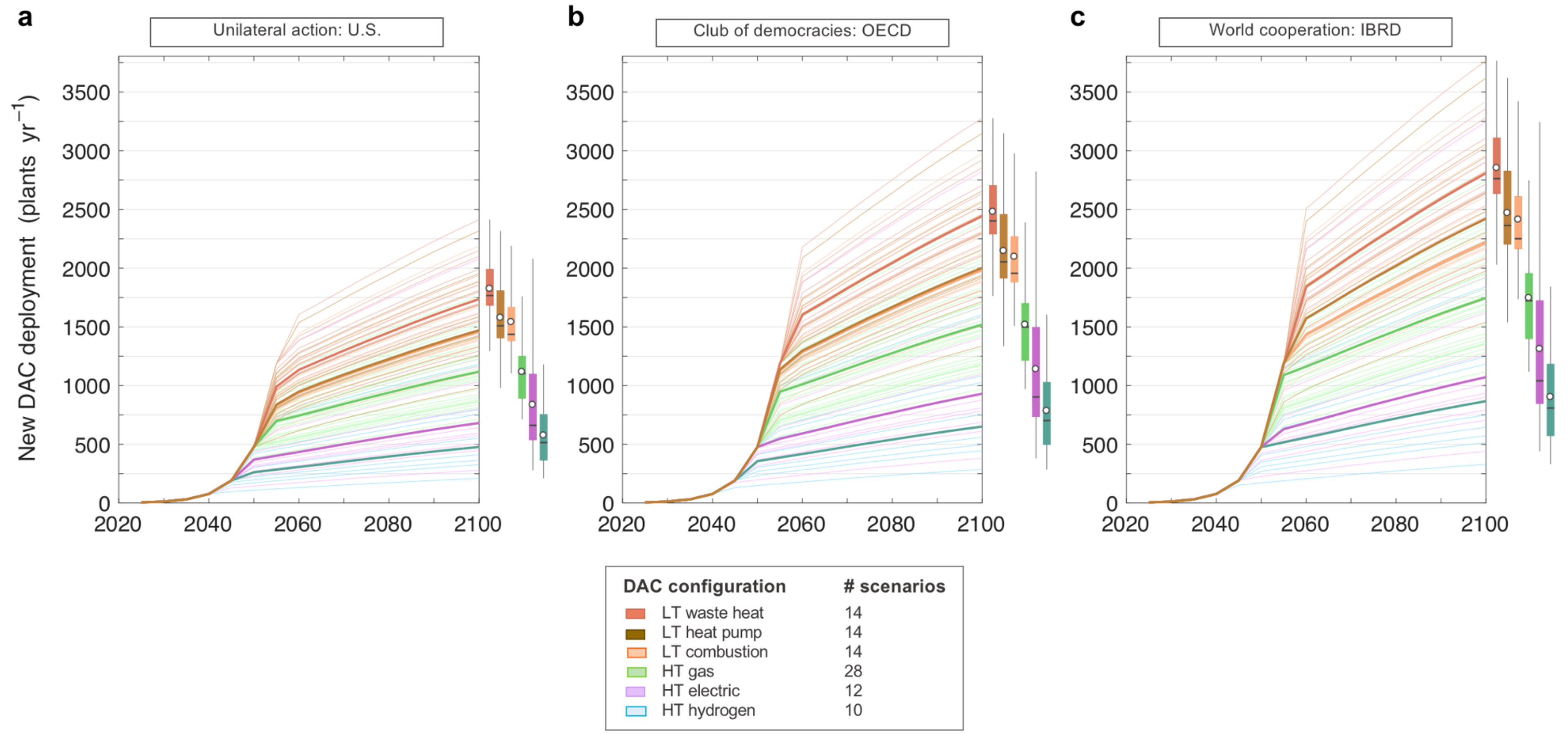

Supplementary Figure 6 | New DAC deployment for individual scenarios by funding regime. a, U.S. unilateral funding. **b,** funding by the club of advanced democracies (OECD members). **c,** world funding (IBRD members). Scenarios with CO_2 removals >0 are shown for the six DAC configurations, which are distinguished by color. Thick lines denote median scenarios. Boxplots show results in 2100: center line, median; dot, mean; box limits, interquartile range; whiskers, range.

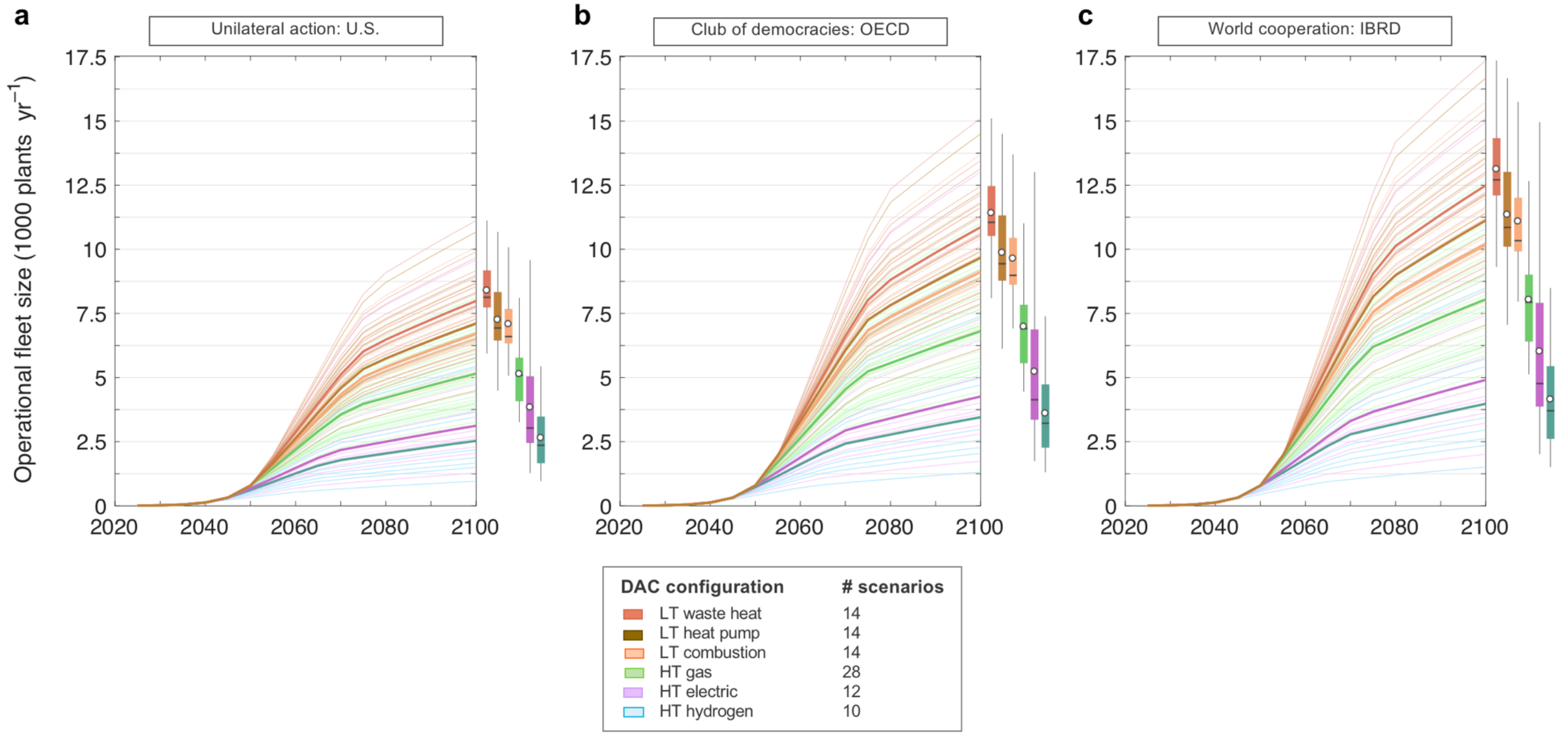

Supplementary Figure 7 | Size of the operational DAC fleet for individual scenarios by funding regime. a, U.S. unilateral funding. **b,** funding by the club of advanced democracies (OECD members). **c,** world funding (IBRD members). Scenarios with CO_2 removals >0 are shown for the six DAC configurations, which are distinguished by color. Thick lines denote median scenarios. Boxplots show results in 2100: center line, median; dot, mean; box limits, interquartile range; whiskers, range.

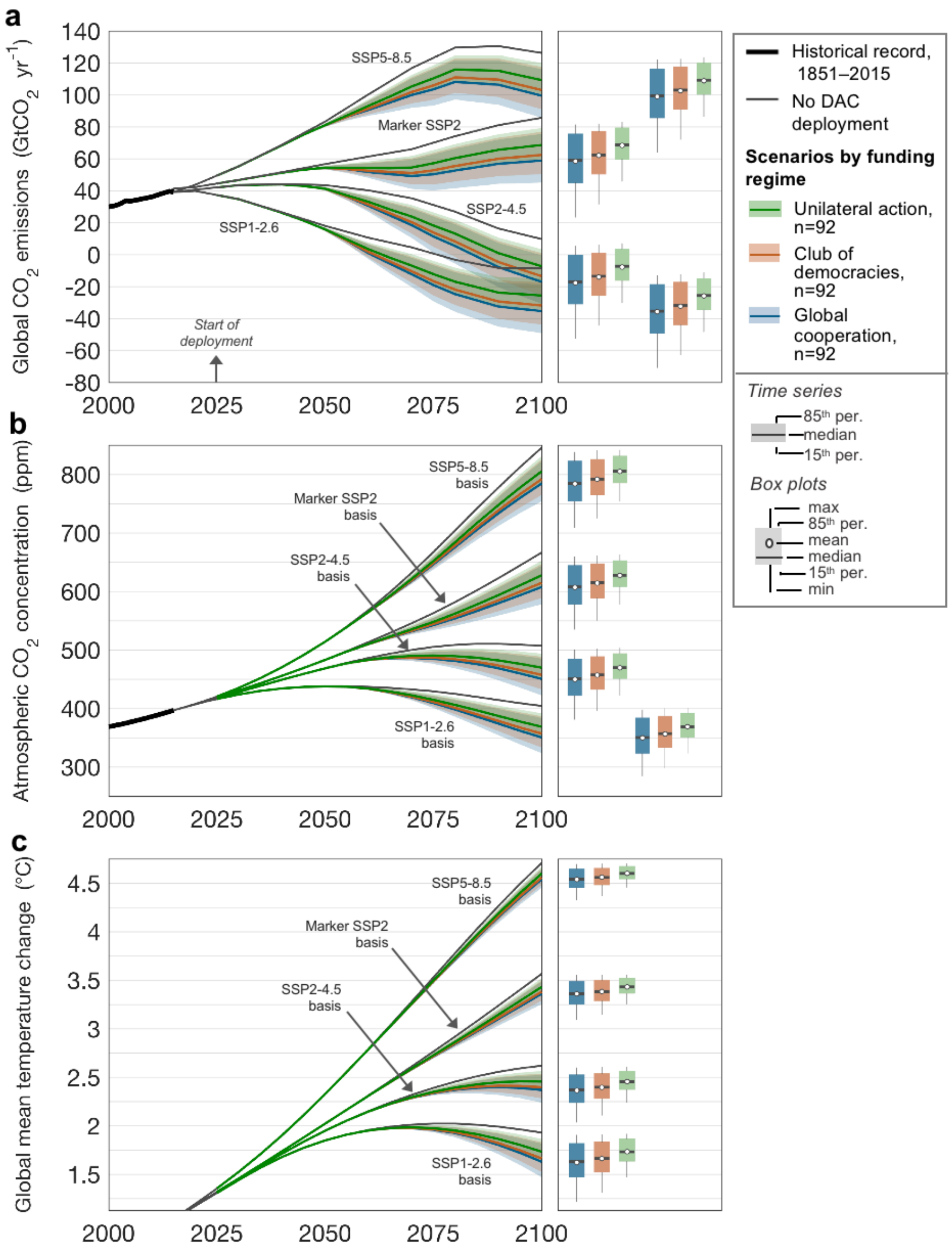

Supplementary Figure 8 | Climate benefits of net CO_2 removal assuming SSP5-8.5, marker SSP2, SSP2-4.5, and SSP1-2.6 emission futures. a, Global CO_2 emissions. DAC deployment commences in 2025. **b,** Atmospheric CO_2 concentration. **c,** Global mean temperature change relative to pre-industrial levels (1850–1900). Ribbons indicate the 15th and 85th percentile scenarios; thick lines indicate the median scenario. Black lines show the case of no DAC deployment[18]. Boxes show the 15th and 85th percentile scenarios in 2100; center line, median; dot, mean; whiskers, range.

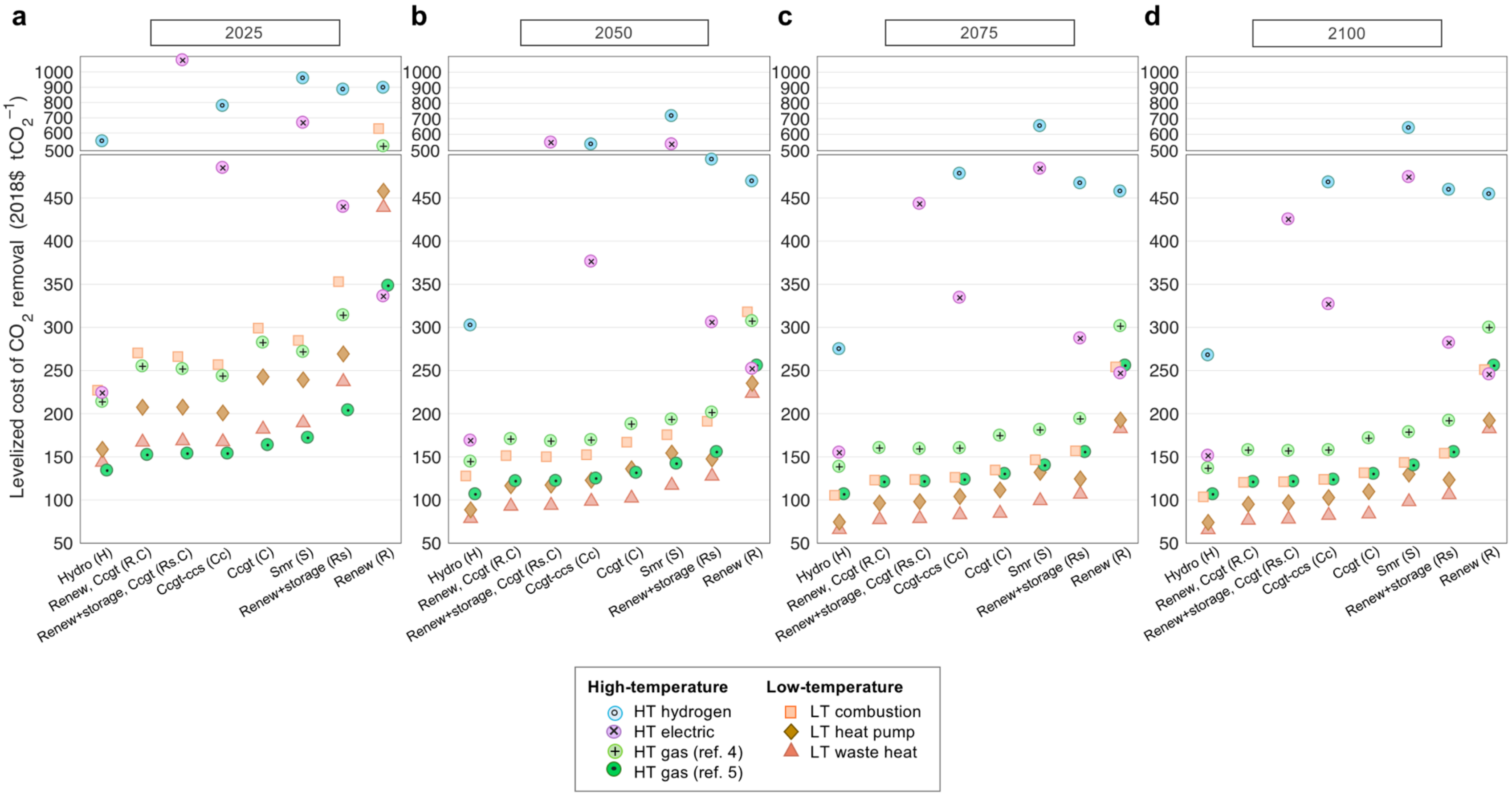

Supplementary Figure 9 | Levelized cost of net CO_2 removal (LCOR) by DAC configuration. Shown are removals by scenario in 2025, 2050, 2075, and 2100 **(a–d)** for scenarios with funding from the club of democracies. DAC configurations (denoted with markers) are plotted by electricity supply (x-axis; Supplementary Table 8). For configurations with energy storage, only the best performing scenario is plotted. For scenarios not shown, process CO_2 emissions exceed removals. Note the break and change in y-axis scaling beginning at 500 2018$ tCO_2^{-1}.

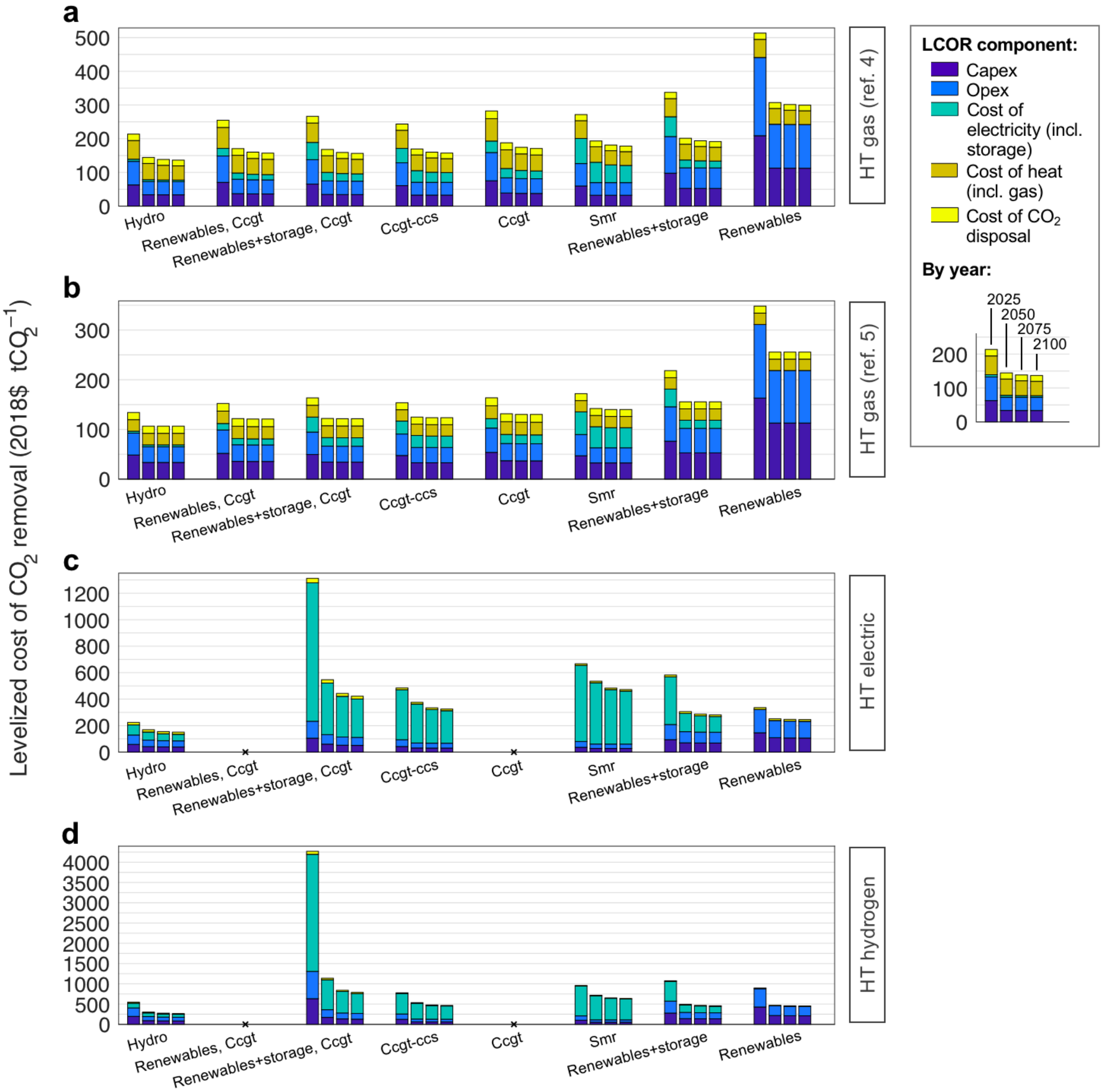

Supplementary Figure 10 | Levelized cost of net CO_2 removal (LCOR) by system component: high-temperature DAC. Shown are the four high-temperature (HT) DAC configurations: HT gas as specified in refs. 4,5 (**a, b**), HT electric (**c**), and HT hydrogen (**d**) shown by electricity source (x-axis; Supplementary Table 8). Bars show LCOR in 2025, 2050, 2075, and 2100. Components include annualized DAC capital and operating costs, energy costs for process heat (inclusive of gas costs) and electricity (inclusive of storage costs), and CO_2 disposal costs. "x" marks denote scenarios where process emissions exceed removals.

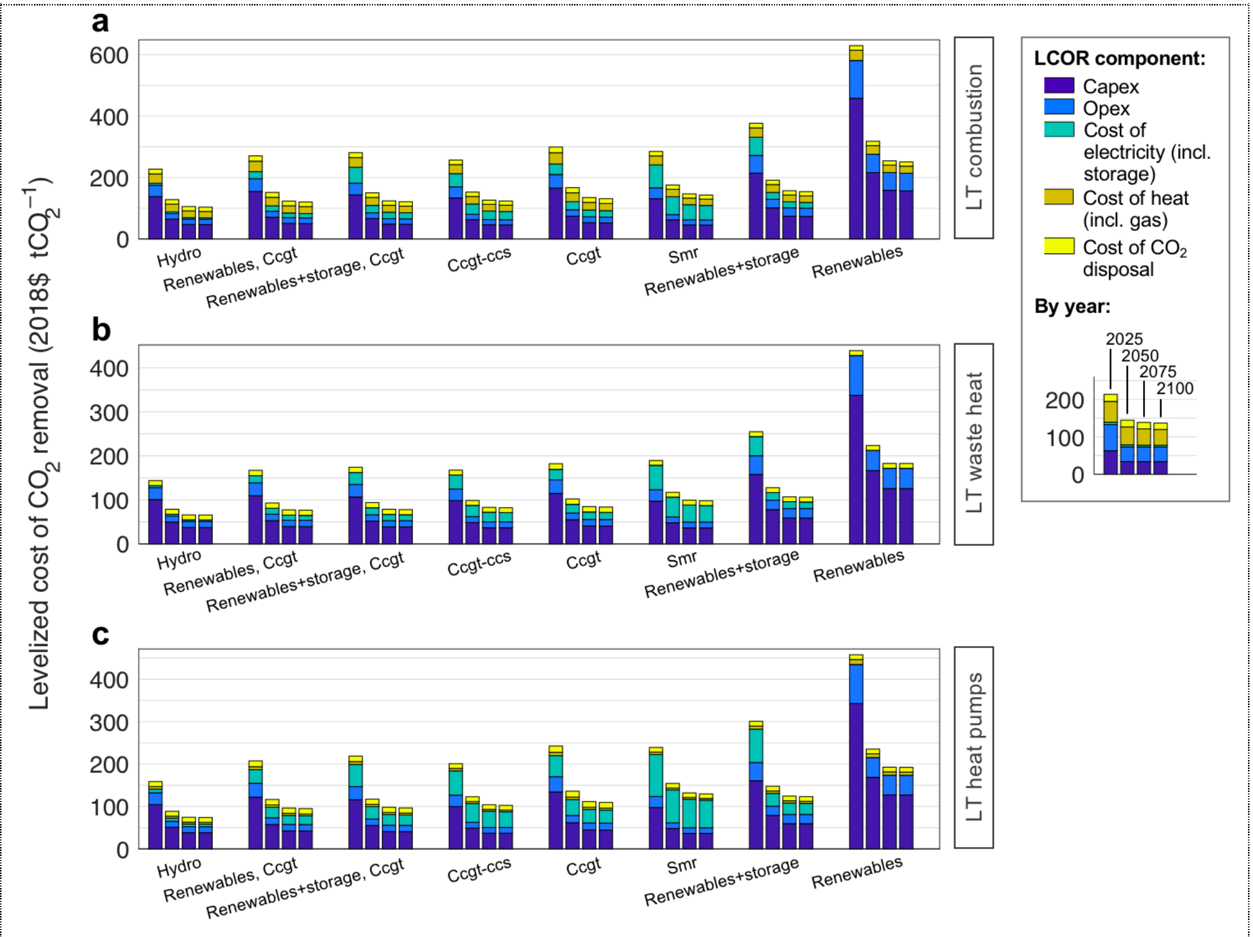

Supplementary Figure 11 | Levelized cost of net CO_2 removal (LCOR) by system component: low-temperature DAC. Shown are the three low-temperature (LT) DAC configurations: LT combustion (**a**), LT waste heat (**b**), and LT heat pumps (**c**) shown by electricity source (x-axis; Supplementary Table 8). Bars show LCOR in 2025, 2050, 2075, and 2100. Components include annualized DAC capital and operating costs, energy costs for process heat (inclusive of gas costs) and electricity (inclusive of storage costs), and CO_2 disposal costs.

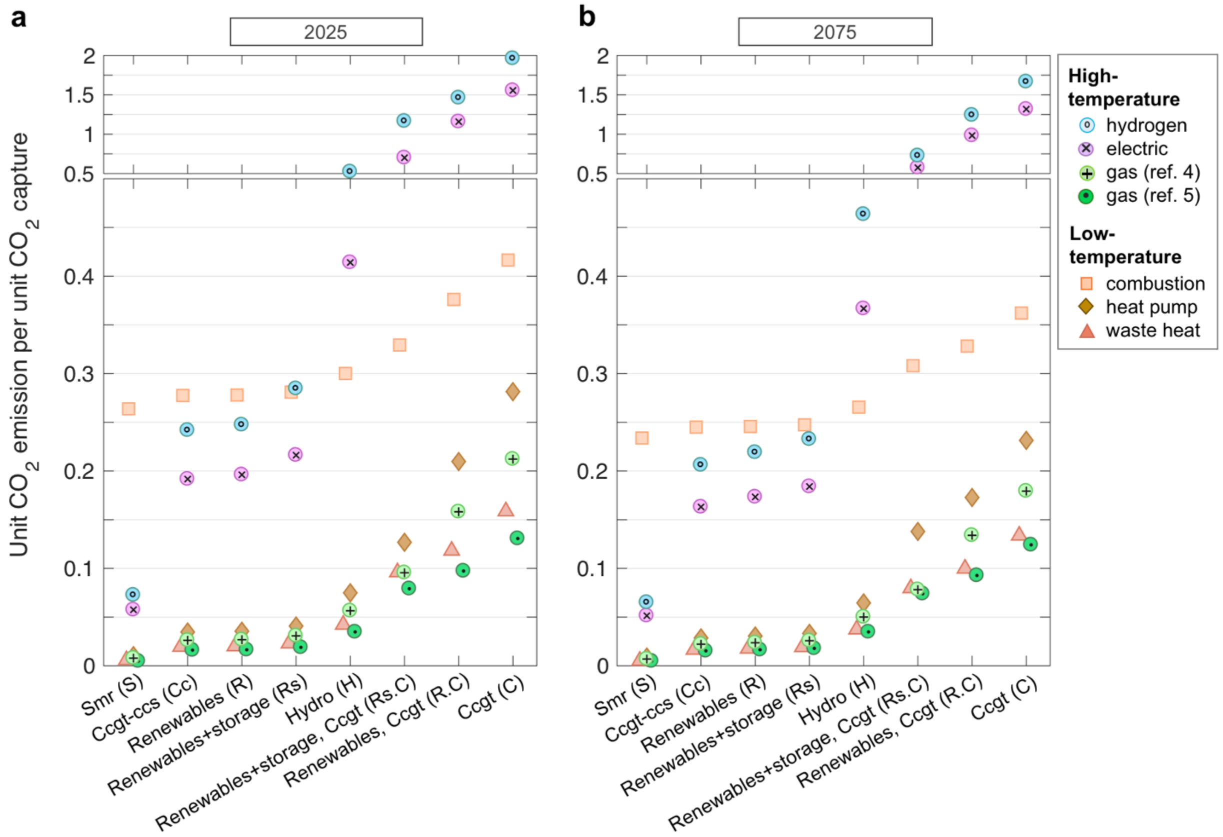

Supplementary Figure 12 | Process emissions by scenario. Process emissions (plotted as the ratio of unit CO_2 emission to unit gross CO_2 capture) by DAC configuration (markers) and electricity supply (x-axis) in 2025 (**a**) and 2075 (**b**) for the case of funding from the club of democracies. Note the break and change in y-axis scaling beginning at 0.5. Ratios >1 imply that use of DAC adds net CO_2 to the atmosphere.

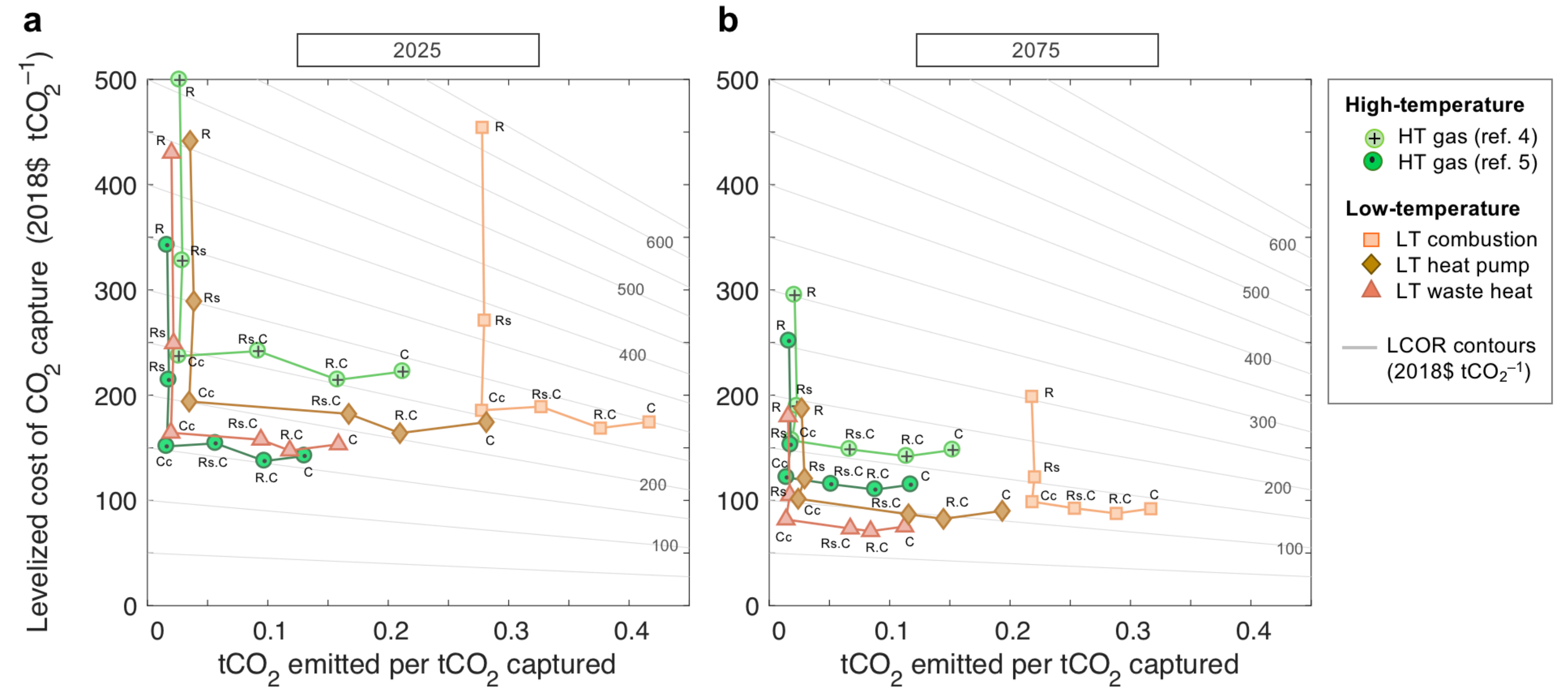

Supplementary Figure 13 | Levelized cost of CO_2 capture, process emissions, and levelized cost of net CO_2 removal (LCOR). Shown are results by scenario in 2025 (**a**) and 2075 (**b**) for select scenarios with funding from the club of democracies. Process emissions (x-axis) are plotted as the ratio of unit CO_2 emission to unit gross CO_2 capture. The levelized cost of *gross capture* (y-axis) is not inclusive of process emissions; the levelized cost of *net removal* (LCOR; contours) is inclusive of emissions. Configurations with high process emissions, such as LT combustion DAC powered with CCGT ("C"), can be cost competitive on the basis of LCOR when their levelized cost of gross capture is low. Electricity labels are consistent with Supplementary Table 8: "R", renewables; "Rs", renewables with energy storage; "C", CCGT without CO_2 capture; "R.C", renewables with CCGT; "Rs.C", renewables with energy storage and CCGT; "Cc", CCGT with CO_2 capture.

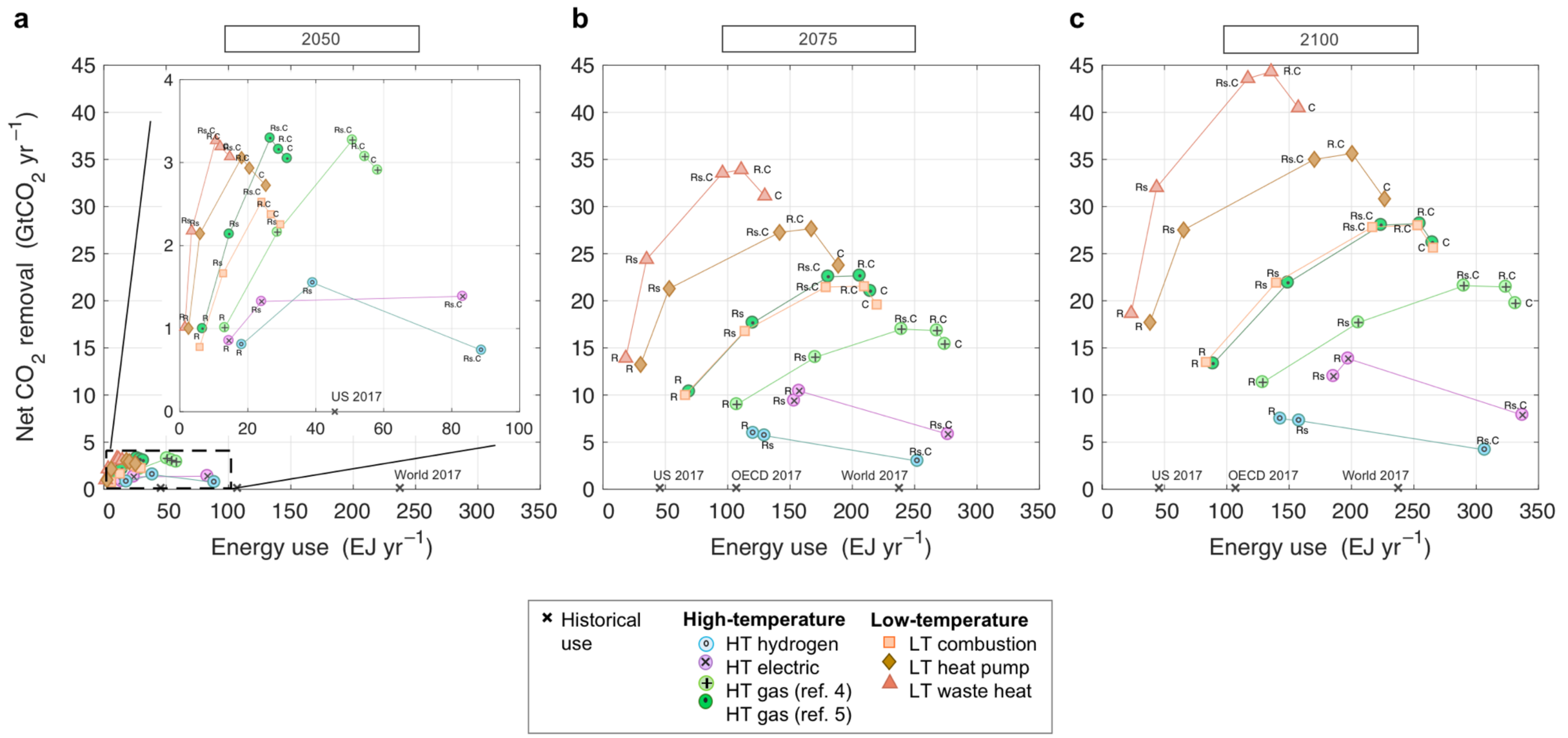

Supplementary Figure 14 | Appraisal of scenarios by net CO_2 removal and energy use. Shown are results for individual scenarios with funding from the club of democracies in 2050, 2075, and 2100 **(a–c).** For configurations with energy storage, only the best performing scenario is shown. Configurations are denoted by markers and electricity supplies by labels. Contours show per-tonne energy use, the ratio of removals to energy use, in GJ tCO_2^{-1}. Electricity labels are consistent with Supplementary Table 8: "R", renewables; "Rs", renewables with energy storage; "C", CCGT without CO_2 capture; "R.C", renewables with CCGT; "Rs.C", renewables with energy storage and CCGT; "H", hydroelectric power; "Cc", CCGT with CO_2 capture; "S", small modular nuclear reactors.

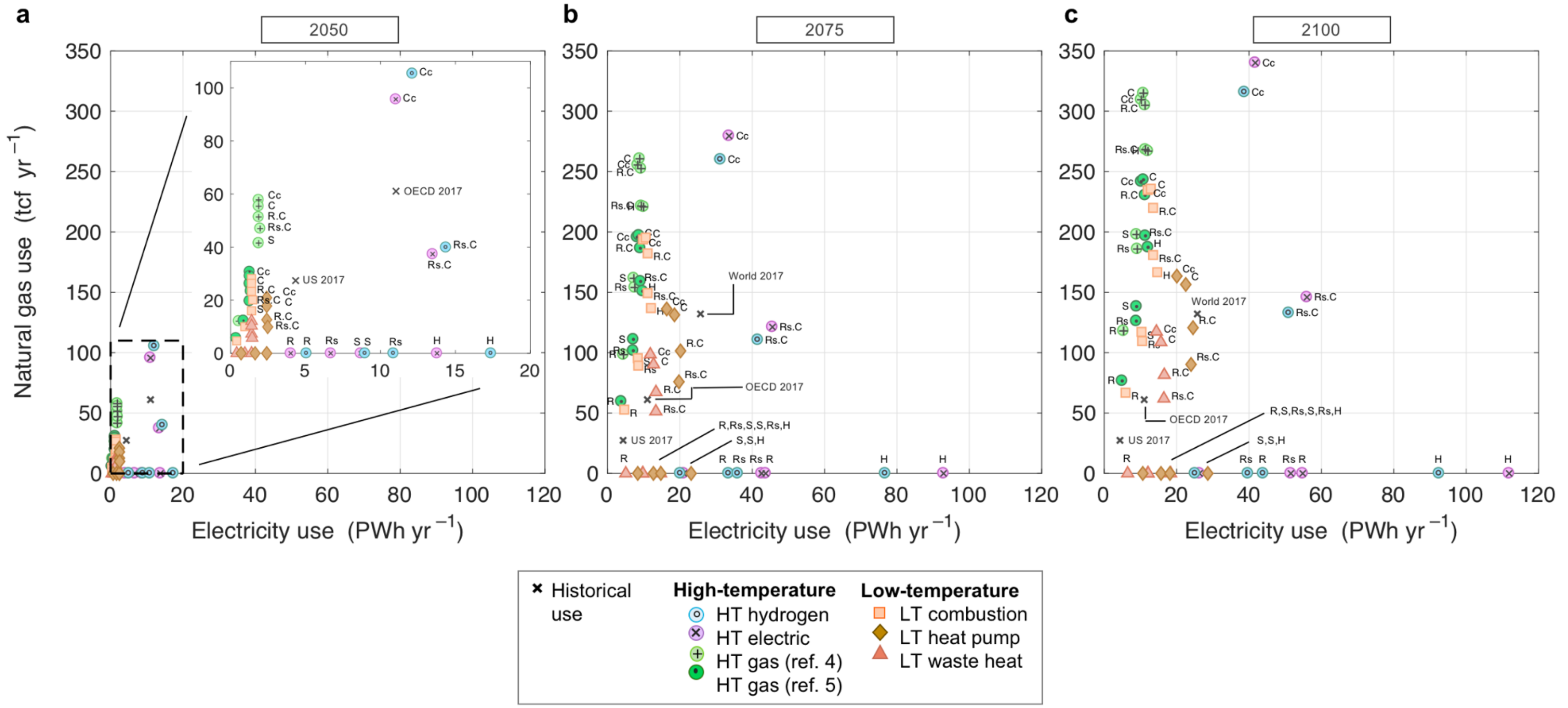

Supplementary Figure 15 | Growth in natural gas and electricity use. Shown are results for individual scenarios with funding from the club of democracies in 2050, 2075, and 2100 **(a–c)**. For configurations with energy storage, only the best performing scenario (largest net CO_2 removal) is shown. Also shown for comparison is 2017 electricity and gas use in the United States, in OECD nations, and globally. Configurations are denoted by marker and electricity sources are denoted with text labels, which are consistent with Supplementary Table 8: "R", renewables; "Rs", renewables with energy storage; "C", CCGT without CO_2 capture; "R.C", renewables with CCGT; "Rs.C", renewables with energy storage and CCGT; "H", hydroelectric power; "Cc", CCGT with CO_2 capture; "S", small modular nuclear reactors. Energy systems require substantial expansion beyond their size today to accommodate a growing DAC fleet. DAC electricity usage exceeds 2017 U.S. electricity by factors of 2–4 and 2017 U.S. natural gas by factors of 2–8 by 2075. (Electric and hydrogen HT DAC and LT systems that use waste heat and heat pumps, which use no gas, are an exception.) Across scenarios there is large variation in gas and electricity use. HT DAC using CCGT (with or without capture) consumes >250 tcf yr^{-1} in 2075, 8-fold larger than the entire U.S. in 2017. By contrast, LT DAC using heat pumps and renewables with storage consumes no gas but rather >30 PWh yr^{-1} electricity in 2075, 6-fold larger than the entire U.S. in 2017. Expansions increase both peak capacity and energy delivery.

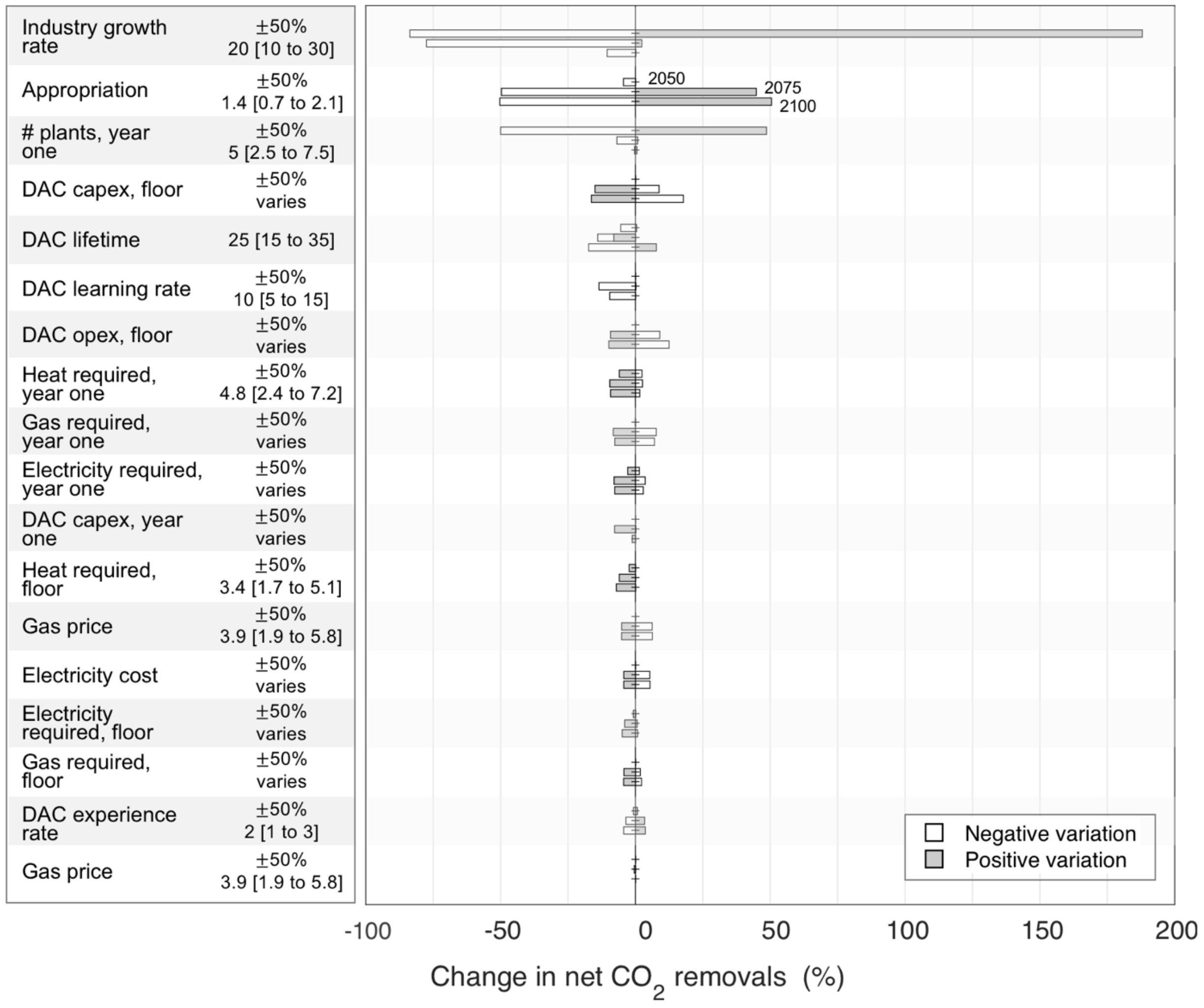

Supplementary Figure 16 | Net CO_2 removal sensitivity to upscaling, DAC plant, and energy system parameters. Bars show the mean change across scenarios in net CO_2 removals in 2050, 2075, and 2100 due to variation in the single parameter. Variation in each parameter (plus/minus and nominal values) is shown at left. White bars show the effect of negative variation (e.g., decreasing growth rate or cost) and gray bars show positive variation. Scenarios included are LT and HT-gas systems with funding by the club of democracies. (HT-electric and HT-hydrogen are outliers relative to LT and HT-gas systems in that they are extremely electricity-intensive and expensive; we omit them here so as not to bias sensitivity on particular parameters, notably electricity required.)

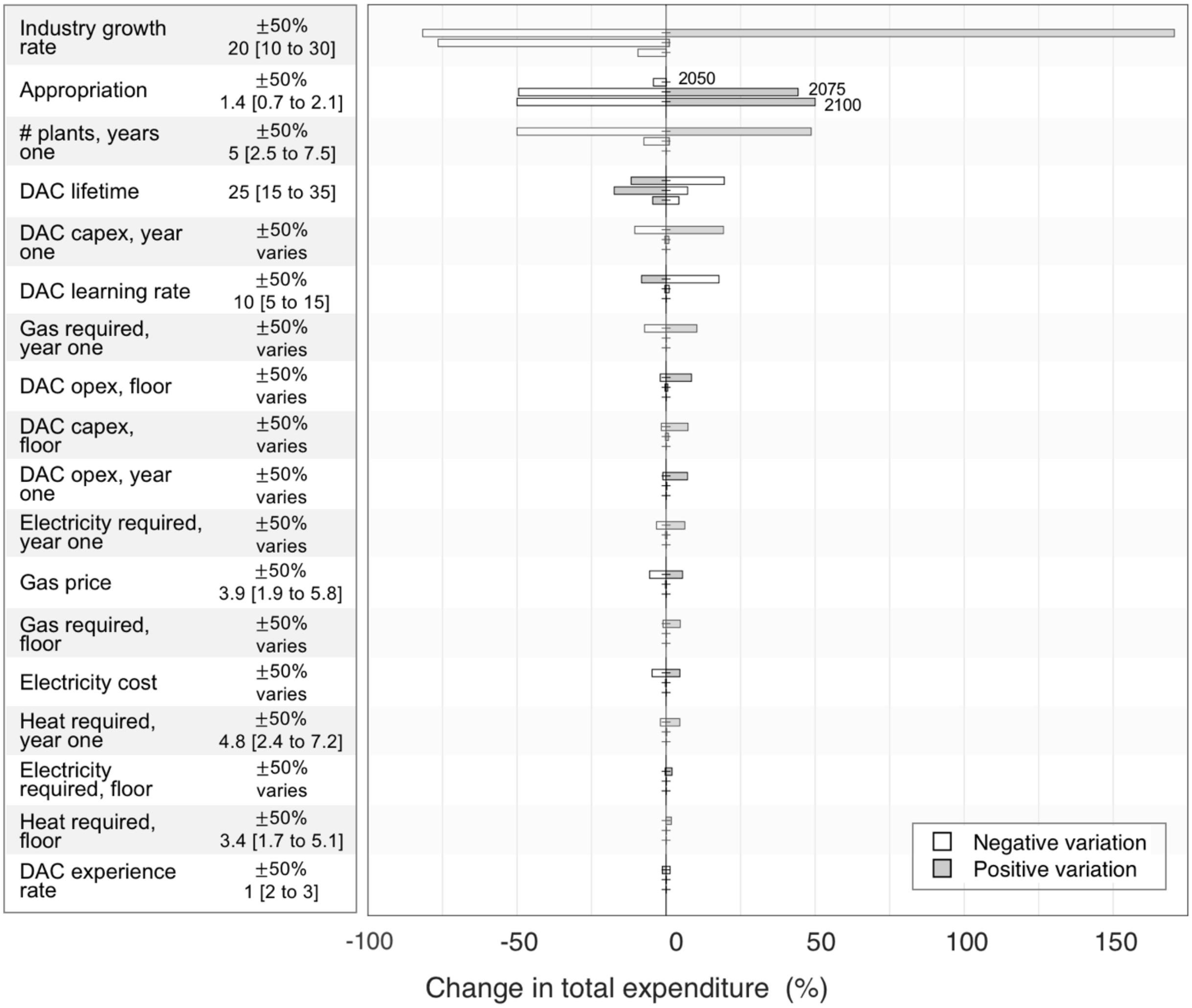

Supplementary Figure 17 | Total expenditure sensitivity to upscaling, DAC plant, and energy system parameters. Bars show the mean change across scenarios in total expenditure on DAC deployment in 2050, 2075, and 2100 due to variation in the single parameter. Variation in each parameter (plus/minus and nominal values) is shown at left. White bars show the effect of negative variation (e.g., decreasing growth rate or cost) and gray bars show positive variation. Scenarios included are LT and HT-gas systems with funding by the club of democracies. (HT-electric and HT-hydrogen are outliers relative to LT and HT-gas systems in that they are extremely electricity-intensive and expensive; we omit them here so as not to bias sensitivity on particular parameters, notably electricity required.)

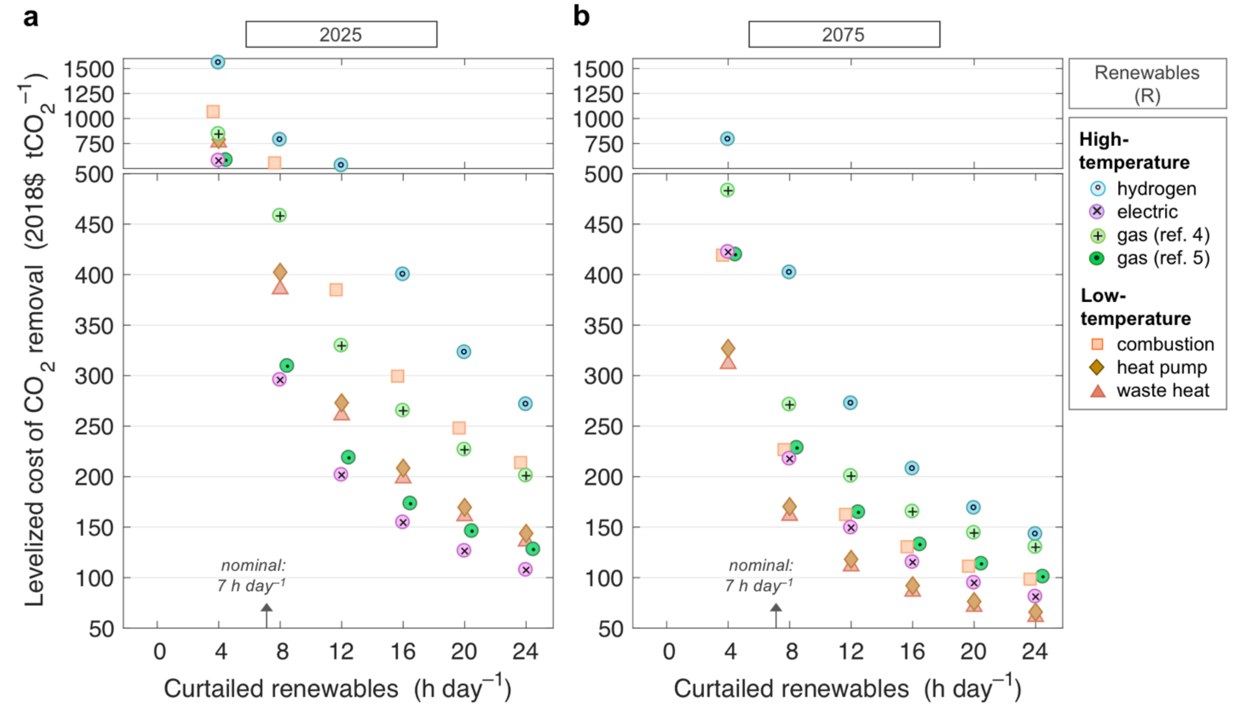

Supplementary Figure 18 | Net CO_2 removal sensitivity to variation in daily hours of renewable power for scenarios with renewables as the electricity supply. Shown are results for the median scenario for the case of funding by the club of democracies. The number of hours used in the base case scenario runs is 7 h day^{-1}.

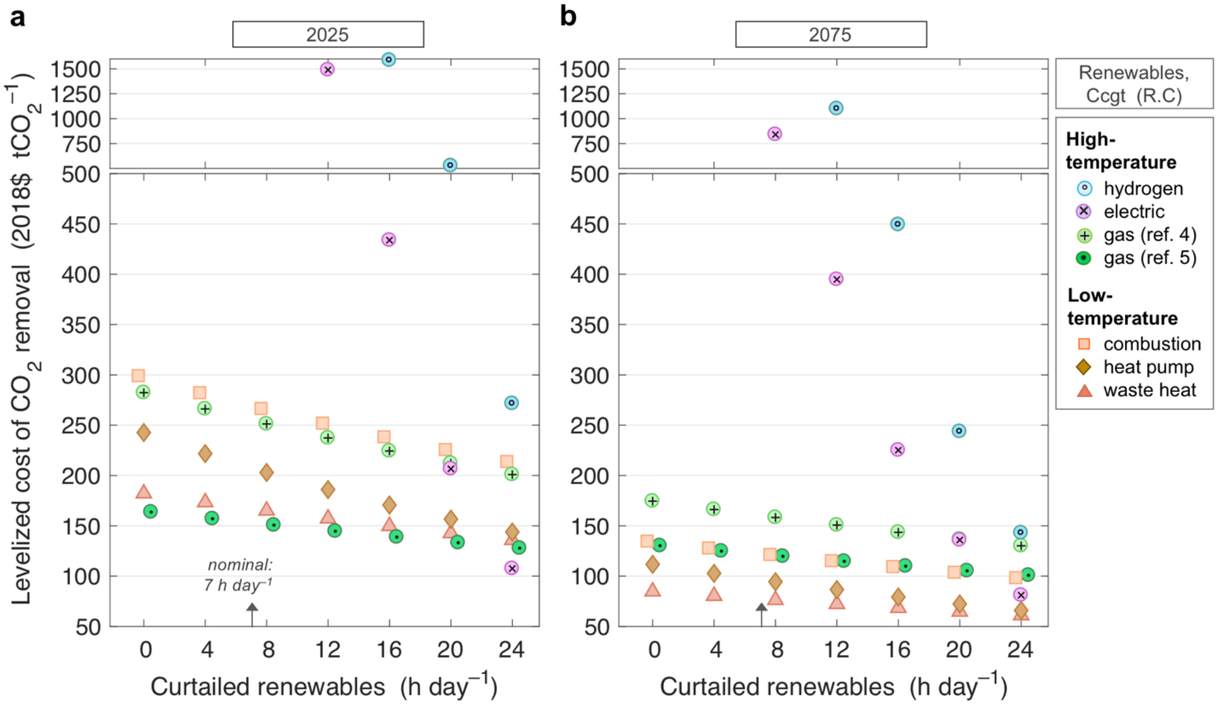

Supplementary Figure 19 | Net CO_2 removal sensitivity to variation in daily hours of renewable power for scenarios with renewables plus CCGT as the electricity supply. Shown are results for the median scenario for the case of funding by the club of democracies. The number of hours used in the base case scenario runs is 7 h day^{-1}.

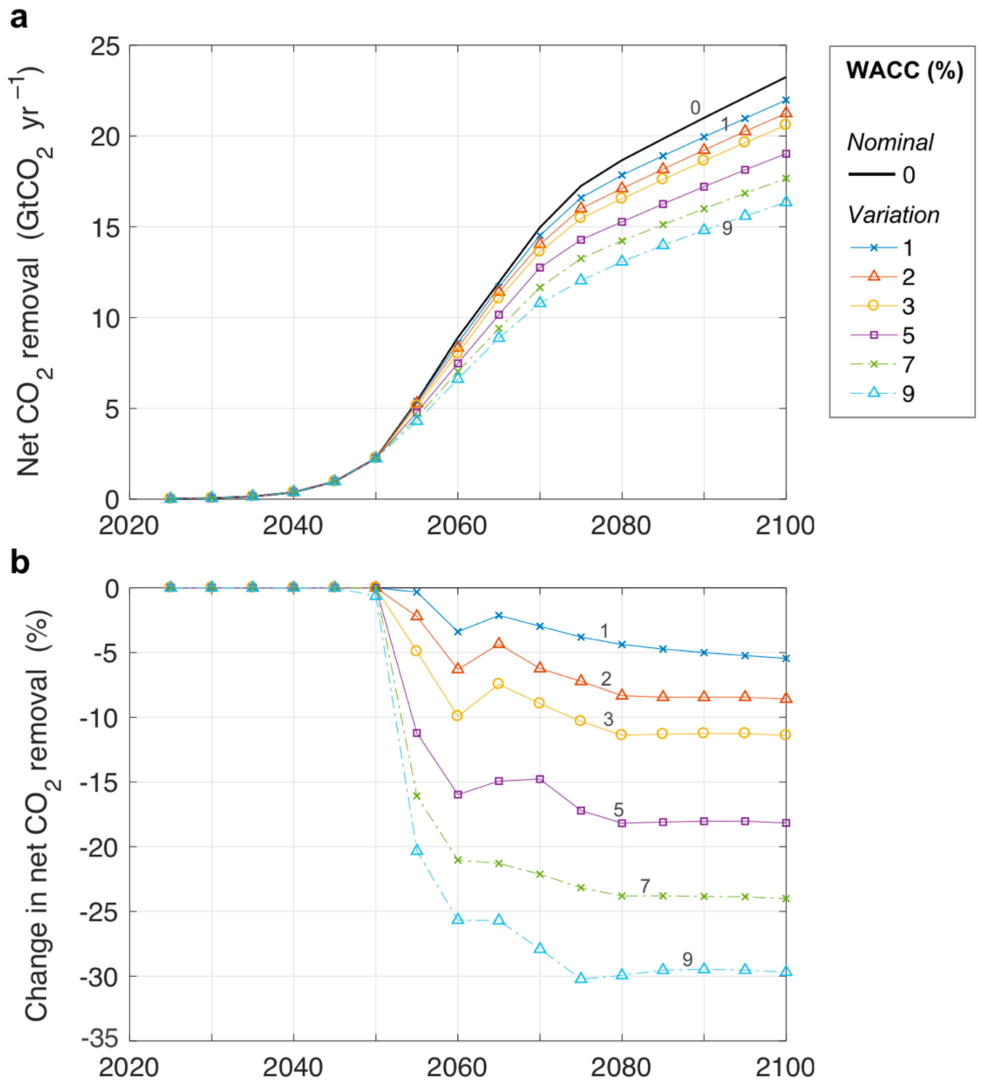

Supplementary Figure 20 | Net CO_2 removal sensitivity to variation in weighted average cost of capital (WACC). a, Net CO_2 removal given variation in WACC. The nominal WAC (0%) is denoted with an unmarked black line; variation is denoted with marked colored lines. Variation covers the full span of U.S. long-term Treasury Bills over the last three decades. **b,** Change in net CO_2 removal relative to the nominal case. Shown are results for the median scenario for the case of funding by the club of democracies.

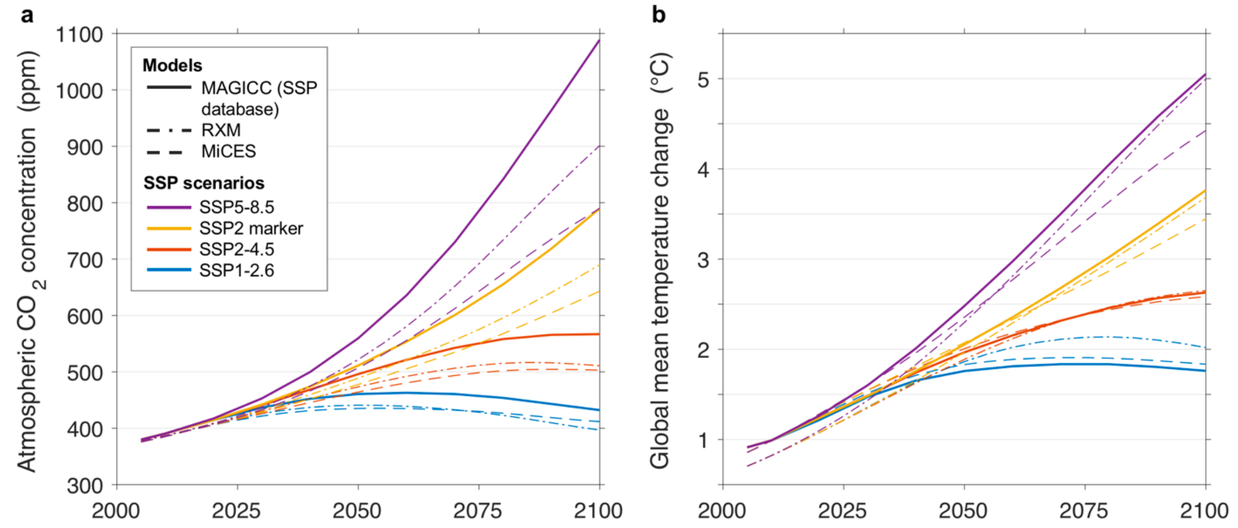

Supplementary Figure 21 | Comparison of climate model output. a, Atmospheric CO_2 concentration. **b,** Global mean temperature rise. Plots show evolution of CO_2 concentration and temperature for the case of no DAC deployment, as output by MAGICC (used in quantification of the SSP scenarios; results taken from the SSP database[18]) and the two models we use in this analysis (RXM, MiCES)[19–23].

Supplementary Table 14 | Effects of delaying deployment of DAC. Presented are results for the scenario of median CO_2 removals for the case of funding by the club of democracies. Base case refers to the central results presented in the paper; the case of 15-year delay considers the identical deployment program but delayed by 15 years to a 2040 start date. "Change" is the percent difference between the two and shows the effect of delaying action. Differences in concentration and temperature are taken considering SSP2-4.5 underlying emissions, are the mean of results from the two climate models we run, and are calculated relative to the case of no mitigation by DAC (not zero).

	**2025 start of deployment (base case)**	**2040 start of deployment (15-year delay)**	**Change**
Rate of annual CO_2 removal, 2050s, $GtCO_2\ yr^{-1}$	1.6 to 4.2	0.1 to 0.3	–94%
Cumulative CO_2 removal, 2025–2100, $GtCO_2$	740	430	–42%
Atmospheric CO_2, 2100, ppm	457	476	38%[a]
Temperature rise above pre-industrial levels (1850–1900), 2100, ºC	2.4	2.5	52%[b]

[a] Calculated relative to the CO_2 concentration in the absence of DAC, for which the average concentration in 2100 across the two climate models is 507 ppm.
[b] Calculated relative to the temperature rise in the absence of DAC, for which the average temperature rise in 2100 across the two climate models is 2.6ºC.

Supplementary References

1. Dellink, R., Chateau, J., Lanzi, E., & Magné, B. Long-term economic growth projections in the Shared Socioeconomic Pathways, *Global. Environ. Chang.* **42**, 200–214 (2017).
2. Leimbach, M., Kriegler, E., Roming, N., & Schwanitz, J. Future growth patterns of world regions – A GDP scenario approach. *Global. Environ. Chang.* **42**, 215–225 (2017).
3. Cuaresma, J.C. Income projections for climate change research: A framework based on human capital dynamics. *Global. Environ. Chang.* **42**, 226–236 (2017).
4. National Academies of Sciences, Engineering, and Medicine. *Negative Emissions Technologies and Reliable Sequestration: A Research Agenda*. (National Academies Press, Washington D.C., 2019).
5. Keith, D. W., Holmes, G., Angelo, D. S., & Heidel, K. A process for capturing CO_2 from the atmosphere. *Joule* **2**, 1573–1594 (2018).
6. National Renewable Energy Laboratory. *Condensing Boilers Evaluation: Retrofit and New Construction Applications*. (NREL, 2014).
7. National Renewable Energy Laboratory. *Electrification futures study: End-use electric technology cost and performance projections through 2050*. Report No. NREL/TP-6A20-70485. (NREL, 2017).
8. Hsu, D.D., *et al*. Life cycle greenhouse gas emissions of crystalline silicon photovoltaic electricity generation. *J. Ind. Ecol.* **16**, S122–135 (2012).
9. Rubin, E.S., Davison, J.E., & Herzog, H.J. The cost of CO_2 capture and storage. *Int. J. Greenh. Gas. Con.* **40**, 378–400 (2015).
10. Carless, T.S., Griffin, W.M., & Fischbeck, P.S. The environmental competitiveness of small modular reactors: A life cycle study. *Energy* **114**, 84–99 (2016).
11. National Nuclear Laboratory. *Small modular reactors (SMR) feasibility study*. (National Nuclear Laboratory, 2014).
12. Schmidt, O., Melchior, S., Hawkes, A., & Staffell, I. Projecting the future levelized cost of electricity storage technologies. *Joule* **3**, 81–100 (2019).
13. Victor, D.G., *et al*. *Pumped energy storage: Vital to California's renewable energy future*. (2019).
14. Brandt, A.R., *et al*. Methane leaks from North American natural gas systems. *Science* **343**, 733–735 (2014).
15. Alvarez, R.A., *et al*. Assessment of methane emissions from the US oil and gas supply chain. *Science* **361**, 186–188 (2018).
16. Barkley, Z. R., *et al*. Forward modeling and optimization of methane emissions in the South Central United States using aircraft transects across frontal boundaries. *Geophys Res Lett* **46**, 13564–13573 (2019).
17. National Renewable Energy Laboratory. *Life cycle assessment of a natural gas combined-cycle power generation*. Report No. NREL/TP-570-27715. (NREL, 2000).
18. Riahi, K., *et al*. The Shared Socioeconomic Pathways and their energy, land use, and greenhouse gas emissions implications: An overview. *Global Environ. Chang.* **42**, 153–168 (2017).
19. Xu, Y. & Ramanathan, V. Well below 2 °C: Mitigation strategies for avoiding dangerous to catastrophic climate changes. *Proc. Natl. Acad. Sci.* **114**, 10315–10323 (2017).
20. Ramanathan, V., & Y. Xu. The Copenhagen Accord for limiting global warming: criteria, constraints, and available avenues. *Proc. Natl. Acad. Sci.* **107**, 8055–8062 (2010).
21. Hu, A., Xu, Y., Tebaldi, C., Washington, W. M., & Ramanathan, V. Mitigation of short-lived climate pollutants slows sea-level rise. *Nat. Clim. Change* **3**, 730–734 (2013).
22. Xu, Y., Zaelke, D., Velders, G. J. M., & Ramanathan, V. The role of HFCs in mitigating 21st century climate change. *Atmos. Chem. Phys.* **13**, 6083–6089 (2013).
23. Chen, J., Cui, H., Xu, Y., & Ge, Q. An Investigation of Parameter Sensitivity of Minimum Complexity Earth Simulator. *Atmosphere* **11**, 95 (2020).
