# Supplementary material for: Emergency deployment of direct air capture as a response to the climate crisis
Source: Nat Commun. 2021 Jan 14;12:368. doi: 10.1038/s41467-020-20437-0 (PMC7809262; doi:10.1038/s41467-020-20437-0)
Supplement: Supplementary file 2 — Reporting Summary [file 41467_2020_20437_MOESM2_ESM.pdf]

nature research

Corresponding author(s): Ryan Hanna

Last updated by author(s): Nov 2, 2020

Reporting Summary

Nature Research wishes to improve the reproducibility of the work that we publish. This form provides structure for consistency and transparency in reporting. For further information on Nature Research policies, see our Editorial Policies and the Editorial Policy Checklist.

Statistics

For all statistical analyses, confirm that the following items are present in the figure legend, table legend, main text, or Methods section.

n/a	Confirmed	
☐	☒	The exact sample size (*n*) for each experimental group/condition, given as a discrete number and unit of measurement
☒	☐	A statement on whether measurements were taken from distinct samples or whether the same sample was measured repeatedly
☒	☐	The statistical test(s) used AND whether they are one- or two-sided<br>*Only common tests should be described solely by name; describe more complex techniques in the Methods section.*
☒	☐	A description of all covariates tested
☒	☐	A description of any assumptions or corrections, such as tests of normality and adjustment for multiple comparisons
☐	☒	A full description of the statistical parameters including central tendency (e.g. means) or other basic estimates (e.g. regression coefficient) AND variation (e.g. standard deviation) or associated estimates of uncertainty (e.g. confidence intervals)
☒	☐	For null hypothesis testing, the test statistic (e.g. *F*, *t*, *r*) with confidence intervals, effect sizes, degrees of freedom and *P* value noted<br>*Give P values as exact values whenever suitable.*
☒	☐	For Bayesian analysis, information on the choice of priors and Markov chain Monte Carlo settings
☒	☐	For hierarchical and complex designs, identification of the appropriate level for tests and full reporting of outcomes
☒	☐	Estimates of effect sizes (e.g. Cohen's *d*, Pearson's *r*), indicating how they were calculated

Our web collection on statistics for biologists contains articles on many of the points above.

Software and code

Policy information about availability of computer code

Data collection	Scenarios were coded and run using Matlab version R2017a.
Data analysis	Analysis of data and results was completed using Matlab version R2017a.

For manuscripts utilizing custom algorithms or software that are central to the research but not yet described in published literature, software must be made available to editors and reviewers. We strongly encourage code deposition in a community repository (e.g. GitHub). See the Nature Research guidelines for submitting code & software for further information.

Data

Policy information about availability of data

All manuscripts must include a data availability statement. This statement should provide the following information, where applicable:

- Accession codes, unique identifiers, or web links for publicly available datasets
- A list of figures that have associated raw data
- A description of any restrictions on data availability

The data that support the findings of this study are available from the corresponding author upon reasonable request.

Field-specific reporting

Behavioural & social sciences study design

All studies must disclose on these points even when the disclosure is negative.

Study description	The study is a systems analysis combining three quantitative datasets: for the direct air capture system, the system of electricity generation, and the system of heat production. These features of three combine in non-trivial ways, so we design the study to include the full universe of scalable combinations.
Research sample	The research sample is the full universe of scalable combinations of direct air capture processes and energy supplies. The features of these systems combine in nontribal ways; as such, we analyze the full universe of scalable combinations. This study uses solely existing datasets, which are provided fully in the supplementary information. Sources include: for direct air capture, academic papers and reports from academia and industry; for energy supplies, technology-specific reports from industry groups, national laboratories, and academics.
Sampling strategy	The sampling procedure sought comprehensiveness. No statistical methods were used to predetermine sample size. One characteristic was required for inclusion among the research sample: that sub-systems (direct air capture or energy supply) be technically scalable to gigaton size. The study does not contain qualitative data.
Data collection	All data was publicly available and retrieved from host sources via the internet. Three of the four authors (R.H., A.A., and D.V.) worked together to identify sources and datasets.
Timing	Data was collected between April and November 2019, without gaps.
Data exclusions	No data were excluded from the analysis.
Non-participation	This study did not use participants.
Randomization	This study did not use participants. Randomization is not applicable to the study because the study is model-based and the model is deterministic once inputs (constituting a scenario) are chosen. Variation in outcomes arises due to the selection of many scenarios, as discussed in sampling strategy.

Reporting for specific materials, systems and methods

We require information from authors about some types of materials, experimental systems and methods used in many studies. Here, indicate whether each material, system or method listed is relevant to your study. If you are not sure if a list item applies to your research, read the appropriate section before selecting a response.

Materials & experimental systems

n/a	Involved in the study
☒	☐ Antibodies
☒	☐ Eukaryotic cell lines
☒	☐ Palaeontology and archaeology
☒	☐ Animals and other organisms
☒	☐ Human research participants
☒	☐ Clinical data
☒	☐ Dual use research of concern

Methods

n/a	Involved in the study
☒	☐ ChIP-seq
☒	☐ Flow cytometry
☒	☐ MRI-based neuroimaging
